# Impaired olfactory performance and anxiety-like behavior in a rat model of multiple sclerosis are associated with enhanced adenosine signaling in the olfactory bulb via A_1_R, A_2B_R, and A_3_R

**DOI:** 10.3389/fncel.2024.1407975

**Published:** 2024-07-30

**Authors:** Andjela Stekic, Milorad Dragic, Jelena Stanojevic, Marina Zaric Kontic, Ivana Stevanovic, Milica Zeljkovic Jovanovic, Katarina Mihajlovic, Nadezda Nedeljkovic

**Affiliations:** ^1^Laboratory for Neurobiology, Department of General Physiology and Biophysics, Faculty of Biology, University of Belgrade, Belgrade, Serbia; ^2^Vinca Institute of Nuclear Sciences, Institute of National Significance, University of Belgrade, Belgrade, Serbia; ^3^Medical Faculty of Military Medical Academy, University of Defense, Belgrade, Serbia; ^4^Department of Molecular Biology and Endocrinology, VINČA Institute of Nuclear Sciences - National Institute of the Republic of Serbia, University of Belgrade, Belgrade, Serbia

**Keywords:** MS/EAE, olfactory impairment, adenosine signaling, A_1_R, A_2B_R, A_3_R, neuroinflammation

## Abstract

The present study shows that animals with experimental autoimmune encephalomyelitis (EAE) exhibit olfactory dysfunction and impaired general cognitive abilities, as well as anxiety-like behavior. Olfactory dysfunction occurs on average at 2 dpi, well before the onset of the first motor signs of EAE (8–10 dpi). After the initial olfactory dysfunction, the EAE animals show a fluctuation in olfactory performance that resembles the relapsing–remitting course of human MS. The study also shows severe neuroinflammation in the olfactory bulb (OB), with numerous infiltrated CD4^+^ T cells and peripheral macrophages in the superficial OB layers, marked microgliosis, and massive induction of TNF-α, IL-1β, and IL-6. Reduced tyrosine hydroxylase activity in the glomerular layer, pronounced granule cell atrophy, and reduced numbers of type B neuroblasts in the rostral migratory stream also indicate altered plasticity of the neuronal network in the OB. Considering the exceptionally high purinome expression in the OB, the possible involvement of purinergic signaling was also investigated. The study shows that macrophages infiltrating the OB overexpress A_3_R, while highly reactive microglia overexpress the adenosine-producing enzyme eN/CD73 as well as A_2B_R, A_3_R, and P2X_4_R. Given the simultaneous induction of complement component C3, the results suggest that the microglial cells develop a functional phenotype of phagocytizing microglia. The study also demonstrates transcriptional and translational upregulation of A_1_R in mitral and tufted cells, which likely influence resting network activity in OB and likely contribute to olfactory dysfunction in EAE. Overall, our study shows that olfactory dysfunction and altered social and cognitive behavior in EAE are associated with increased adenosine signaling via A_1_R, A_2B_R, and A_3_R.

## 1 Introduction

Multiple sclerosis (MS) is a chronic, immune-mediated, inflammatory, and neurodegenerative disease of the central nervous system (CNS) that primarily affects young adults (Greer and McCombe, 2011). It is characterized by autoimmune attacks on components of the myelin sheath, leading to demyelination and axonal loss (Filippi et al., 2018) and ensuing motor and cognitive disabilities (Mandolesi et al., 2010). In most patients, the disease takes a relapsing–remitting course (RRMS), which develops into a secondary progressive form (SPMS) over time, while in a smaller number of patients, the disease takes a progressive course from the start (PPMS). In both cases, the chronic course of the disease worsens other neurological abilities, including mental health and cognition. As a result, MS leads to early retirement and permanent disability for those affected, which places an enormous socio-economic burden on society.

Although the main neurological manifestations of MS include motor disability and impaired cognition, transient sensory dysfunctions, known as clinically isolated syndrome (CIS), typically occur in the early prodromal phase of the disease, long before full-blown symptoms develop. Impaired vision, facial tactile, and hearing loss may occur in the prodromal phase (Christogianni et al., 2018); however, anosmia is one of the most common CIS manifestations observed retrospectively in MS patients (Lucassen et al., 2016; Kim et al., 2018). Recent meta-analyses show a prevalence of 25–45% olfactory dysfunction in MS patients (Lucassen et al., 2016; Mirmosayyeb et al., 2022), with a recent study showing that the degree of olfactory dysfunction positively correlates with a severity of the disease (Todd et al., 2023). Since early deterioration of olfactory function is also characteristic of other neurodegenerative diseases such as Parkinson’s disease (PD), Alzheimer’s disease (AD), and Lewy body disease (Slabik and Garaschuk, 2023), full knowledge of the mechanism of olfactory dysfunction could enable the use of olfactory performance as a diagnostic marker or as a marker of the efficacy of intervention or therapy.

**Figure 1:**
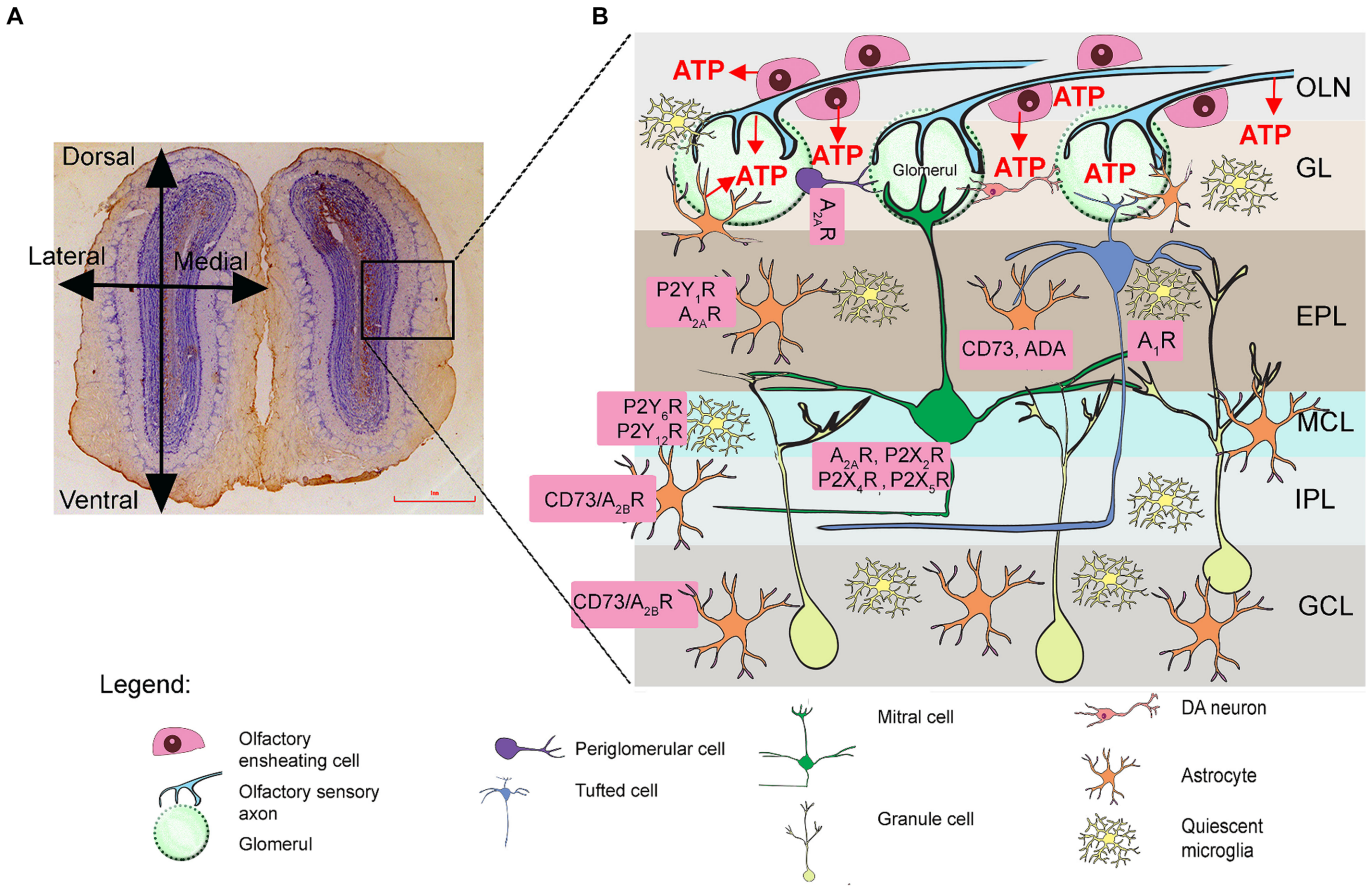
Purinergic signaling in the olfactory bulb (OB). **(A)** Schematic representation of the OB tissue section (*left*) and schematic representation of the OB (*right*). The frame shows the part enlarged in the drawing in **(B)**, showing the distribution of puronoreceptors and ectonucleotidases in the OB cellular network, based on physiological, immunological, and histochemical data (see Rotermund et al., 2019). ATP is released from sensory axon terminals and periglomerular astrocytes in the ONL and GL. Adenosine A_1_R and A_2A_R as well as P2X_2_R/P2X_4_R/P2X_5_R were identified in the mitral cells and tufted cells. The expression of P2Y_1_R and A_2A_R was detected in astrocytes in the GL, EPL, and MCL. Unusually high activity and expression of eN/CD73 and adenosine deaminase (ADA) were detected in the EPL, IPL, and GCL. GCL, granule cell layer; GL, glomerular layer; EPL, external plexiform layer; IPL, internal plexiform layer; MCL, mitral cell layer; ONL, olfactory nerve layer.

Recent studies show that the blood–cerebrospinal fluid (CSF) barrier (Strominger et al., 2018) and the CSF-filled subarachnoid space are the initial sites where immune cells infiltrate the subpial and periventricular spaces of the brain and spinal cord in EAE (Shin et al., 2019; Lazarevic et al., 2023). Several studies conducted in experimental autoimmune encephalomyelitis (EAE), the predominant animal model for MS (Constantinescu et al., 2011; Bjelobaba et al., 2018), have found altered odor-related behaviors and histopathological changes in olfactory bulb tissue in diseased animals (Kim et al., 2018; Schubert et al., 2022). However, the underlying mechanism of olfactory dysfunction and the contribution of neuroinflammation are still unclear. Sensation of smell begins in the olfactory sensory neurons (OSN), which are specialized bipolar neurons embedded in the olfactory epithelium at the roof of the nasal cavity. The axons of the OSN enter the cranial cavity through the holes of the cribriform plate and terminate in the olfactory bulb (OB) (Figure 1). These short, non-myelinated axons are surrounded by olfactory ensheathing cells (OEC), a special type of glial cell which together form the olfactory nerve layer (ONL) (Field et al., 2003). The sensory axon terminals enter the glomerular layer (GL), where they synapse on the apical dendrites of mitral and tufted cells in the so-called glomeruli (Nagayama et al., 2014). The glomeruli also include various types of interneurons and astrocytes that surround the glomerular neuropil. The cell bodies of the tufted neurons are located in the external plexiform layer (EPL), while the mitral cell bodies form a single-row mitral cell layer (MCL) directly below the EPL. Mitral cells have large pyramidal cell bodies and apical dendrites that terminate in a single glomerulus, while their lateral dendrites branch into the EPL, where they form reciprocal dendro-dendritic synapses with granule cells and local interneurons. Immediately below the MCL, the axons of the mitral and tufted cells gather and extend caudally through the internal plexiform layer (IPL), forming the lateral olfactory tract (LOT). The deepest OB layer is the granule cell layer (GCL), occupied by small axonless granule interneurons that project their dendrites into the EPL. The medial part of the OB consists of the subependymal layer (SE), which comprises the tip of the rostral migratory stream (RMS). The RMS is a migratory route along which migrating type A neuroblasts that originated in the subventricular zone (SVZ) of the brain migrate to reach the OB, surrounded by specialized type B cells that form glial tubes that provide scaffolds for the migration (Nagayama et al., 2014).

Purinergic signaling plays an important role in sensory transduction and information flow through the olfactory system and is also involved in inflammation (Burnstock and Kennedy, 2011; Fredholm, 2014; Rotermund et al., 2019; Santiago et al., 2020; Pietrowski et al., 2021). ATP is released in the olfactory epithelium or co-released with glutamate from the axon terminals of the OSN in the glomeruli and ectopically along the axons in the ONL (Rotermund et al., 2019). Another important source of ATP are astrocytes, which constitutively release ATP via connexin 43 hemichannels (Roux et al., 2015) and contribute by 50% to the unusually high extracellular ATP levels in the OB. ATP acts upon P2 receptors, of which P2X_2_R, P2X_4_R, P2X_5_R, and P2X_6_R have been detected in mitral and tufted cells (Rotermund et al., 2019), while they are absent in glial cells (Collo et al., 1996; Vulchanova et al., 1997; Guo et al., 2008; Kaneda et al., 2010), which in contrast abundantly express the P2Y_1_R receptor subtype (Doengi et al., 2008). Extracellular ATP is rapidly hydrolyzed by ectonucleotidase enzyme chain that terminates its signaling activity. ATP is hydrolyzed to ADP or directly to AMP by ecto-nucleoside triphosphate diphosphohydrolase 2 (NTPDase2) and ecto-nucleoside triphosphate diphosphohydrolase 1 (NTPDase1/CD39), respectively, while ecto-5′-nucleotidase (eN/CD73) completes nucleotide hydrolysis by forming adenosine. Adenosine can be further degraded by adenosine deaminase (ADA) or transported back into the cells by equilibrative (ENT) or concentrative (CNT) nucleoside transporters (Zimmermann and Braun, 1999). The localization and activity of individual ectonucleotidases varies between the different OB layers but is significantly higher compared to other brain regions, indicating rapid extracellular ATP turnover and the importance of adenosine signaling in this sensory system (Langer et al., 2008). Particularly high activity has been demonstrated for eN/CD73 and ADA in the EPL, IPL, and GCL (Langer et al., 2008), which are by far the highest within the whole rodent brain. As for NTPDase1/CD39 and NTPDase2, both ectonucleotidases show strong expression in the EPL and IPL and weak expression in the GL (Rotermund et al., 2019), with NTPDase2 being very strongly expressed in the RMS (Shukla et al., 2005; Gampe et al., 2015).

While the degradation of other signaling molecules leads to their inactivation, purinergic signaling is unique in that the products of ATP hydrolysis also function as signaling molecules. Specifically, ADP acts at P2Y_6_R and P2Y_12_R, which are mainly located in microglia (Chen et al., 2018), while adenosine acts at four subtypes of P1 receptors that are coupled to inhibition (A_1_R, A_3_R) or activation (A_2A_R, A_2B_R) of adenylate cyclase (Fredholm et al., 2011). While all adenosine P1 receptors are found in the OB, A_1_R, and A_2A_R are among the most abundant throughout the rodent brain (Rotermund et al., 2019). A_1_R is found in mitral and tufted cells (Kaelin-Lang et al., 1999), while A_2A_R is found in periglomerular interneurons and astrocytes as well as granule cells (Rotermund et al., 2018). A recent study has shown that A_1_R plays an important role in the modulation of dendro-dendritic synapses between mitral and granule cells and influences the processing of olfactory information (Rotermund et al., 2018; Schubert et al., 2022).

Purinergic signaling also plays a special role in neuroinflammation when ATP is released in large quantities from neurons and astrocytes in inflamed tissue. Under such conditions, ATP primarily activates the low-affinity P2X_7_R (Domercq et al., 2019), which triggers the assembly of inflammasomes in microglia and astrocytes and the secretion of inflammatory cytokines (for a review, see Di Virgilio et al., 2023). Microglia and astrocytes in the affected tissue upregulate NTPDase1/CD39 and eN/CD73, which enable increased ATP turnover and efficient production of adenosine (Lavrnja et al., 2009; Lavrnja et al., 2015; Jakovljevic et al., 2019). Although adenosine is generally considered an anti-inflammatory molecule (Cunha, 2005), under such conditions it activates A_2A_R, which mediates inflammatory response in reactive astrocytes and microglia (Cunha, 2016). Due to the exceptionally high expression of purinergic receptors and nucleotide-degrading enzymes in the OB and the involvement of purinergic signaling in neuroinflammatory processes associated with MS/EAE, in the present study, we mainly focused on adenosinergic signaling in the OB in rats with induced EAE.

## 2 Materials and methods

### 2.1 Animals

The study was performed on 2-month-old male Dark Agouti (DA) rats (Supplementary Table S1). Animals were obtained from the animal facility of the Military Medical Academy, University of Defense, Serbia (RRID: RGD_21409748). All experimental procedures complied with ARRIVE guidelines and were performed in accordance with EU Directive 2010/63/EU. The Ethics Committee for the Care and Use of Laboratory Animals of the Faculty of Biology of the University of Belgrade issued a positive opinion under the number 119-01-4/11/2020-09. All animals were kept under standard conditions with 12-h light/dark cycles at constant ambient temperature (22 ± 2°C) and humidity. All other conditions were kept constant in all experimental units and throughout the study to avoid performance bias.

### 2.2 Induction of EAE and neurological assessment

Experimental autoimmune encephalomyelitis was induced by intradermal injection of 150 μL of an encephalitogenic emulsion into the left hind paw. The encephalitogenic emulsion was prepared by mixing spinal cord homogenate [1 mg tissue/μl phosphate-buffered saline (PBS)] with the same volume of complete Freund’s adjuvant (CFA) supplemented with 4 mg/mL *Mycobacterium tuberculosis*, as previously described (Dragić et al., 2021a). Prior to immunization, animals were anesthetized by intraperitoneal injection of ketamine (50 mg/kg) and xylazine (10 mg/kg). Nothing was injected into the hindpaw of control rats.

The animals were daily weighed and scored according to the clinical EAE severity scale for motor disability (Bachmann et al., 1999): 1—limp-tailed, 1.5—clumsy, 2—mild monoparesis or paraparesis, 3—hind paw paralysis, 3.5—paraplegia or/and quadriparesis, 4—quadriplegia, 5—moribund state or death. Animals were sacrificed on average at 12–14 dpi when they reached the EAE score of 2.5–3, which corresponded to the state of paraplegia and/or quadriparesis (Figure 1B). The control group consisted of age-matched animals that underwent the same experimental protocol, except for the encephalitogenic injection. Complete epidemiological data are summarized in Supplementary Table S1.

### 2.3 Buried food test

Olfactory performance was analyzed by a buried food test (Kim et al., 2018; Machado et al., 2018). The test was performed daily, starting at −3 dpi until sacrifice. Animals had no access to food during the 17 h preceding the testing (17:00–08:00 h). The body mass was monitored daily to assure that testing did not impact their overall health condition. The feeding regimen resulted in <5% loss of the initial body mass (Supplementary Table S1). Each morning, the animals (*n* = 10 per experiment repeated three times) were brought into the experimental room, where they were allowed to adapt for 60 min. Each animal was placed in the experimental arena (box 50 cm × 50 cm × 50 cm, 3 cm thick bedding) and allowed to rest for 2 min. The animal was then moved to the clean cage while the pellet was buried 0.5 cm below the bedding in any corner of the test arena. The animal was again placed in the test arena, and activity was recorded for 5 min in dim light (>100 lux) with an ANY-MAZE camera. After each test, the bedding was changed, and the arena was cleaned with 70% ethanol for the next test. Animals that did not show typical exploratory behavior in BFT, which means they did not move from one spot of the arena during the trial at all, were excluded from the study.

The recorded material was analyzed after each measurement. For each animal, the average latency to find buried food, determined from −3 dpi to 0 dpi, was referred to as the baseline latency (s). The time taken by an animal to find buried food from 0 dpi afterwards was expressed relative to its baseline latency, i.e., each animal was a control to itself.

### 2.4 Olfactory discrimination task

Odor discrimination was tested at 3 dpi and 7 dpi in the odor discrimination task (ODT), with slight modifications (Soffié and Lamberty, 1988). Baseline performance was determined for each animal (*n* = 10) on the day before immunization (−1 dpi). The rats were placed in the test room and had 60 min to adapt. The test arena consisted of two identical compartments (50 cm × 25 cm × 50 cm) connected by a passageway (10 cm × 10 cm). One compartment contained sawdust from the cage in which the tested animals had spent the last 24 h (familiar odor), while the other compartment contained sawdust from a cage of an unrelated group of animals (unfamiliar odor). The activity of the animal was recorded for 5 min with the ANY-MAZE camera under dim light, and the time (min) the animal spent in each compartment (familiar vs. unfamiliar) was measured. After each trial, the sawdust in the test arena was changed, and the sides of the test arena were cleaned with 70% ethanol. As a parameter for ODT, the discrimination index (DI) represents the difference in exploration time between the two compartments (unfamiliar compartment – familiar compartment) divided by the total amount of exploration in both compartments (unfamiliar compartment + familiar compartment) × 100 (%). DI values can range from −100 to 100%. The DI value close to zero indicates that the animal explores both compartments equally, which indicates loss of odor discrimination ability. The opposite is to be expected if an animal with intact olfactory function prefers to explore a particular compartment. Animals that tend to explore unfamiliar odors have high positive DI values, while animals that tend to spend more time in the compartment with the familiar odor have negative DI values. Data are presented as mean DI (±SD) from one determination in three experimental units.

### 2.5 Open field test

Spontaneous behavior was analyzed in the open field arena at 7 dpi and was compared to baseline behavior determined at −1 dpi. The animals (*n* = 12) were habituated to a new environment for 2 h. Each animal was placed in the center of the empty black arena (100 × 100 × 50 cm), and activity was recorded continuously for 10 min using the ANY-MAZE camera under dim light (>100 lux). The sides and floor of the test arena were cleaned with 70% ethanol after each session. The following parameters were extracted by digitally analyzing the recorded video material: number of entries into central/peripheral fields/corners, time spent in central/peripheral fields/corners, distance traveled in central/peripheral fields, latency to enter central field, average speed in central/peripheral fields/corners, maximum speed in central/peripheral fields/corners, mobile time spent in central/peripheral fields/corners, immobile time spent in peripheral fields/corners, number of immobile episodes in peripheral fields/corners, number of freezing episodes in peripheral fields/corners and freezing time spent in peripheral fields/corners (freezing time was calculated if the animal was not moving for more than 10 s, while if the animal was not moving for more than 30 s it was considered as immobility).

### 2.6 Novel object recognition test

The impact of neuroinflammation on general cognitive ability was tested with novel object recognition test (NORT) at 7 dpi. Each animal (*n* = 12) was placed in the center of the testing arena at the position equidistant from two identical rectangular objects (uniformly yellow-colored, diameter 5 × 5 × 20 cm). The animal was left to freely explore objects for 10 min and then returned to their home cages (sampling phase). For the testing phase, one object was replaced with a new one (uniformly red-colored conical object, 15 × 20 cm, *d* × *h*). The animal was returned to the test arena after 3 h and left to freely explore for another 10 min (testing phase). Animal activity was recorded with ANY-MAZE camera under dim light (>100 lux). After each session, both arena and objects were carefully cleaned with 70% alcohol to ensure the complete removal of any olfactory cues which could interfere with the testing. Percentage of time spent exploring the novel object relative to the total time spent exploring both objects is expressed as recognition index (RI) and was used to assess novel object recognition (Antunes and Biala, 2012).

### 2.7 Immunohistochemistry and confocal immunofluorescence

The animals (*n* = 4/group) were sacrificed by decapitation. Brains were carefully dissected, fixed in 4% paraformaldehyde/0.1 phosphate buffer (pH 7.4) for 24 h, and cryoprotected/dehydrated in 10, 20, and 30% sucrose–phosphate buffer. Serial 25-μm coronal brain sections at stereotaxic coordinates Bregma 7.56–6.60 mm (Paxinos and Watson, 2013) were cut on a cryostat, mounted on SuperFrost^™^ Plus adhesion microscope slides (Epredia Inc., United States), air-dried at RT for 1–2 h, and stored at −20°C until use.

For immunohistochemical staining, sections were left at RT for 30 min, rehydrated in PBS (pH 7.4) for 2 × 5 min, and incubated with 0.3% H_2_O_2_/methanol at RT for 20 min to inhibit endogenous peroxidase activity. After washing in PBS for 2 × 5 min, the sections were blocked with 5% normal donkey serum (NDS)/PBS for 1 h at RT. The sections were then incubated overnight with the primary antibodies at 4°C (Supplementary Table S2). After washing in PBS for 3 × 5 min, sections were incubated for 2 h at RT with HRP-conjugated secondary antibodies (Supplementary Table S2). 3,3-S-diaminobenzidine tetrachloride (DAB kit, Abcam, UK) was used as a chromogen for HRP-conjugated secondary antibodies. The reaction was stopped in PBS. Sections were incubated with thionin for 20 min at RT to counterstain the tissue. Sections were dehydrated in 50, 70, 95, and 100% ethanol for 5 min each, cleared in xylene for 2 × 5 min, and mounted with DPX medium (Sigma Aldrich, United States). Digital images (2,088 × 1,550 pixels) were acquired using LEITZ DM RB light microscope (Leica Mikroskopie & Systems GmbH, Wetzlar, Germany), LEICA DFC320 CCD camera (Leica Microsystems Ltd., Heerbrugg, Switzerland), and LEICA DFC Twain software (Leica, Germany).

For immunofluorescence staining, sections incubated with primary antibodies were incubated with fluorophore-labeled secondary antibodies (Supplementary Table S2) for 2 h at RT in a dark chamber. Sections were washed in PBS for 3 × 5 min and mounted with Mowiol (Sigma Aldrich, USA). Images (1,024 × 1,024) were captured using a confocal laser scanning microscope (LSM 510, Zeiss) with an argon laser (488 nm) and two helium–neon lasers (534 nm and 633 nm) at 40× or 63× magnification.

### 2.8 Quantification of immunohistochemistry, immunofluorescence, and multi-image colocalization analysis

A number of Iba1^+^ and TH^+^ cells were counted in low-power micrographic images (40×) using the ImageJ Cell Counter plugin.^1^ The number of immunopositive cells was counted in the corresponding OB layers in *n* = 7 sections per experimental group and expressed as a number of cells/mm^2^. High-resolution images (1,024 × 1,024) were analyzed for integrated density in the JACoP plugin (Bolte and Cordelieres, 2006), and the degree of overlap between multiple signals was assessed by calculating the Pearson correlation coefficient (Dunn et al., 2011). The Pearson correlation coefficient (PSS) is a statistical parameter that reflects the co-occurrence of pixels from different channels, regardless of the intensity levels and signal offset. Seven to nine images/animal (four animals/experimental group) were acquired with the same laser gain and exposure. PCC analysis was performed at 40× or 63× magnification. Whole images were used for analysis. Prior to colocalization analysis, confocal images were adjusted by background correction (Landmann and Marbet, 2004).

### 2.9 Gene expression analysis

Total RNA was extracted from OB tissue using TRIzol reagent (Invitrogen, Carlsbad, CA, United States), according to the manufacturer’s instructions. The concentration and purity of RNA were determined using OD260 and OD260/OD280, respectively. Complementary DNA (cDNA) was synthesized using the High-Capacity cDNA Reverse Transcription Kit (ThermoFisher Scientific, Waltham, MA, United States) according to the manufacturer’s instructions. Quantitative real-time PCR was performed by the ABI Prism 7000 Sequence Detection System (Applied Biosystems, United States) by mixing cDNA with primers (Supplementary Table S3) and Fast EvaGreen qPCR Mastermix (Applied Biological Materials Inc., Richmond, BC, Canada), under the following conditions: 10 min enzyme activation at 95°C, 40 cycles of 15 s denaturation at 95°C, 30 s annealing at 60°C, 30 s amplification at 72°C, and 5 s fluorescence measurement at 72°C. Samples from five animals/experimental group were run in duplicate. The relative expression of target genes was quantified using the 2^−ΔCt^ method relative to CycA, which was used as a reference gene. Data were expressed as fold-change of gene expression in EAE compared to control. At the end of each experiment, a melting curve analysis was performed to confirm the formation of a single PCR product.

### 2.10 Preparation of crude membrane fraction (P2 fraction)

Crude membrane fraction (P2) was isolated from OB tissue by using a previously established protocol (Gray and Whittaker, 1962). Briefly, OB tissue (g) was homogenated in nine volumes (ml) of ice-cold isolation buffer (0.32 M sucrose in 5 mM HEPES pH 7.4) supplemented with 0.5% w/v protease inhibitor cocktail (ab65621, Abcam, UK). The crude nuclear fraction (P1) was pelleted by centrifugation at 1,000 × *g* for 10 min, while centrifugation of the resulting supernatant at 16,000 × *g* for 30 min yielded the plasma membrane fraction (P2). The P2 pellet was resuspended in HEPES buffer, and the samples were stored at −20°C until use. Protein content was determined using the Micro BCA Protein Assay Kit (Thermo Fisher Scientific, Rockford, United States). Samples were aliquoted and stored at −80°C until use.

### 2.11 Western blot analyses

**Figure 2:**
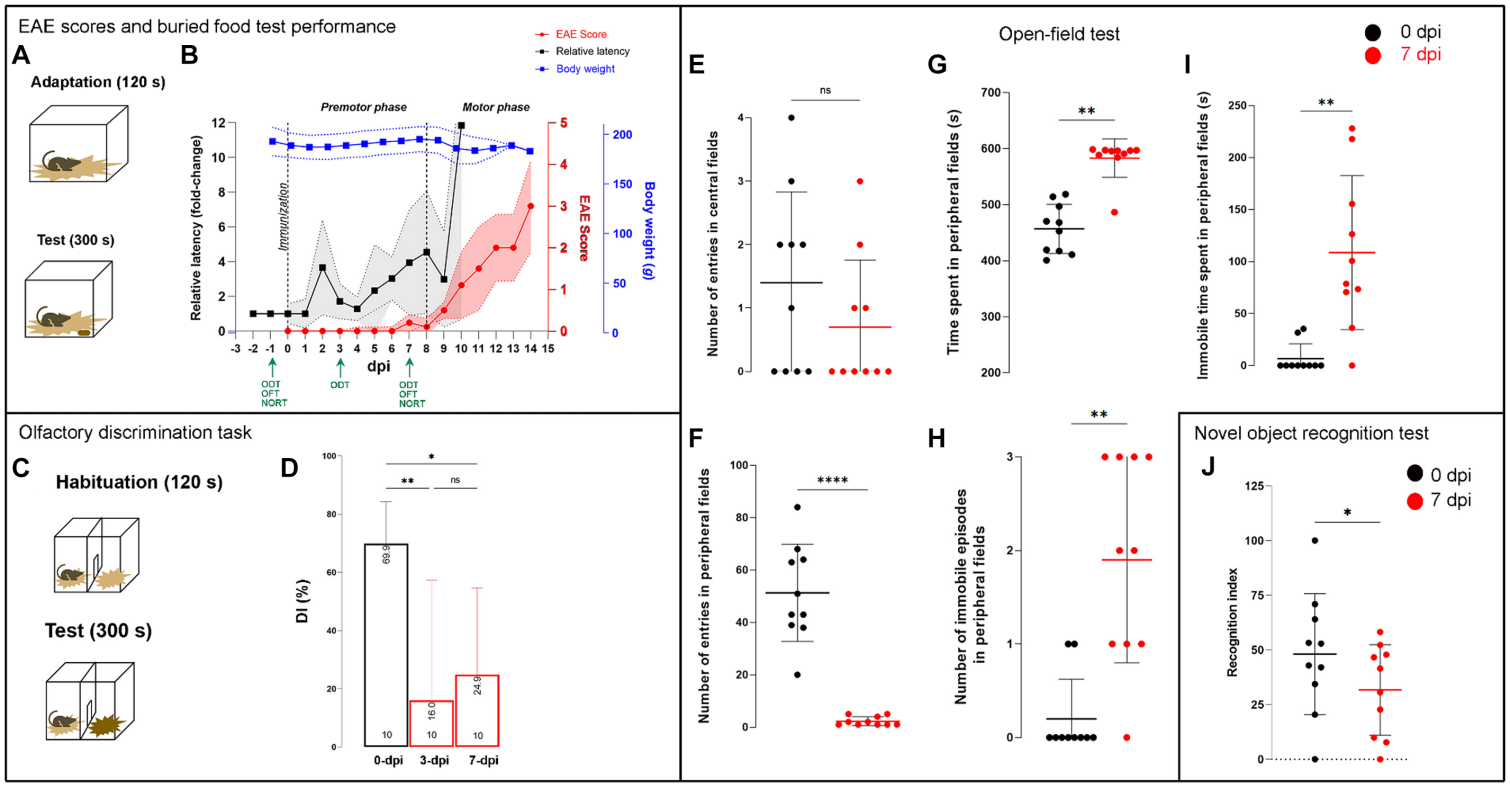
Neurological data and behavioral testing in EAE. **(A)** Schematic representation of the buried food test protocol, consisting of a 120-s adaptation and a 300-s test series. **(B)** The timeline shows the changes in neurological scores (*red circles*), average body mass (*blue squares*), and relative latency (*black squares*) over the course of the EAE (dpi). Data are presented as mean ± SD (colored area). The dashed lines represent the day of immunization (0 dpi) and the day of onset of motor symptoms (8 dpi), which determine the duration of the premotor phase of EAE. Green arrows indicate the days on which the behavioral tests were performed. **(C)** Schematic representation of the olfactory discrimination test protocol and **(D)** variations in the discrimination index (DI), which represents the difference between the time spent in the unfamiliar and familiar compartments divided by the total time spent in both compartments × 100 (%). The olatfactory discrimination test was performed at −1, 3, and 7 dpi with three experimental units, and the data are expressed as mean DI (±SD). **(E–I)** Parameters from the open field test performed at −1 and 7 dpi with 3 experimental units, and data are expressed as mean ± SD. **(J)** Recognition index (RI), calculated in novel object recognition test, as the time spent with a new object relative to the total time spent exploring both objects × 100 (%). The test was performed at −1 dpi and 7 dpi with three experimental units, and the data are presented as mean (±SD). Significance within the graphs: *p < 0.05; **p < 0.01; ***p < 0.001.

P2 samples adjusted to 1 mg/mL in 6× Laemmli buffer [4% sodium dodecyl sulfate (SDS), 0.02% bromophenol blue, 20% glycerol, and 125 mmol/L Tris–HCl] were boiled at 95°C for 5 min. About 20-μl aliquots were separated on 10% SDS-polyacrylamide gels and electrotransferred to 0.45 μm PVDF support membranes (Millipore, Germany) using the Trans-Blot Turbo Transfer System (Bio-Rad, Hercules, CA, United States). The support membranes were blocked for 1 h at RT with 5% non-fat dry milk (SERVA, Germany) in Tris-buffered saline containing 0.1% Tween-20 (TBST) and then incubated overnight at 4°C with the primary antibodies diluted in TBST (Supplementary Table S2). After washing in TBST for 3 × 5 min, the immunoreactivity profile was detected by incubating the support membranes at RT for 1 h with HRP-conjugated secondary antibodies diluted in TBST (Supplementary Table S2) and rinsing in TBST for 3 × 5 min at 4°C using the SmartBlot device. The chemiluminescent signal was detected with ECL solution (Bio-Rad, Hercules, CA, United States) using the ChemiDoc-It Imager (Ultra-Violet Products Ltd., Cambridge, United Kingdom). Primary and secondary antibodies were removed using a mild stripping protocol (0.2 mmol/L glycine, 0.1% SDS, and 1% Tween-20, pH 2.2) to blot the GAPDH, which was used as a loading control. The abundance of the target protein was quantified by densitometry using ImageJ software (NIH, Bethesda, MA, United States). The optical density (OD) of the target protein band was normalized to the OD of the GAPDH band in the same lane, and the ratio was expressed relative to the control sample (defined as 100%). Data were expressed as mean target protein abundance ± SD (%) of four samples/group blotted two times. Cropped PVDF support membranes presented in Figure 2 are presented integrally in Supplementary Figure S2.

### 2.12 Ectonucleotidases enzyme assays

Ectonucleotidase activities were determined by measuring the amount of inorganic phosphates (Pi) released by enzymatic hydrolysis of ATP, ADP, or AMP (Nedeljkovic et al., 2003), using the malachite green protocol as previously described (Grković et al., 2019). About 10-μl aliquots of the P2 sample (1 mg/mL protein concentration) were pre-incubated at 37°C in the assay buffer (10 mM HEPES pH 7.4 and 1 mM MgCl_2_) for 10 min (for ATP and ADP hydrolysis) or 30 min (for AMP hydrolysis). The reaction was started by adding 1 mM nucleotide solution to the assay buffer. The reaction was stopped by adding 22.5 μL of 3 M perchloric acid. 20 μL of malachite green working solution (0.1% malachite green in 20% H_2_SO_4_, 7.5% ammonium molybdate, and 11% Tween-20 in a ratio of 10:2.5:0.2) was added to 80 μL of the reaction mixture and incubated at RT for 30 min to form a green-colored complex whose absorbance was measured at 620 nm. The amount of Pi released was calculated using the calibration curve generated with KH_2_PO_4_ as standard. The results were expressed as mean activity (nmol Pi/mg/min) ± SD, determined in four different P2 samples per experimental group, performed two times in duplicate.

The activity of adenosine deaminase (ADA) was determined by measuring the amount of NH_4_^+^ ions released by the enzyme reaction (Dragic et al., 2022). Aliquots containing 20 μg of the P2 sample proteins were pre-incubated for 10 min at 37°C and the reaction was started by adding 1 mM adenosine. The reaction was carried out for 2 h at 37°C and stopped by adding 500 μL phenol nitroprusside reagent and 500 μL sodium hypochlorite reagent. After 15 min at 37°C, the absorbance, which is directly proportional to the NH_4_^+^ concentration, was measured at 635 nm. The amount of NH_4_^+^ was calculated from the calibration curve using NH_4_Cl as a standard. The results were expressed as mean activity (nmol NH_4_^+^/mg/h) ± SD, determined on four different P2 preparations per group, performed two times in duplicate.

### 2.13 Study design and statistics

A total of 90 animals were divided into 22 experimental units (4–5 animals/cage), of which 17 experimental units (66 animals) were randomly assigned to the EAE group, while the remaining five (18 animals) formed the control group (Supplementary Table S1). The sample size was estimated using G*Power analysis (Faul et al., 2007). All animals were weighed daily and examined for motor signs of EAE. Each animal was sacrificed on the day it had an EAE score of 2.5–3, which was an average at 12 dpi. In addition to weighing and EAE scoring, each experimental unit was used only for one particular behavioral test prior to sacrifice and preparation of OB samples (microscopic sections, mRNA extracts, and P2 membrane fractions). Samples from animals belonging to one experimental unit were not pooled and used only for one particular analysis. Each behavioral test and molecular analysis was repeated minimum of two times with different animals.

Quantitative data obtained within the study fell into the category of continuous data, with the exception of neurological scores, which were discrete. Data were analyzed using descriptive statistics and expressed as mean ± SD or SEM, as indicated in figure legends. The normality of data was tested using the Shapiro–Wilk test, and then appropriate parametric or non-parametric tests were used. For EAE to control group comparisons (gene expression level, WB, hydrolysis rate, PCC), the two-tailed Student’s *t*-test was used or Wilcoxon signed rank test if the normality condition was not met. Values of *p* < 0.05 were considered statistically significant. Neurological assessments (BFT, ODT, OFT, and NORT) were performed as repeated measurements in the EAE group only, and the collected data were compared within-subject and between-group. Differences were assessed using a repeated-measures ANOVA followed by a Dunnett’s *post-hoc* test, with the significance level set at 0.05 or with Mann–Whitney *U* test if the normality condition was not met, while for between-group comparison, the two-tailed Student’s *t*-test was used.

Relevant biases were identified, and methods were applied to reduce them. Confounding factors were mitigated by randomization to experimental units with equal and predetermined sample sizes sufficient to ensure statistical power without compromising the data quality or robustness of the study. Performance biases were attenuated by standardization and constant conditions in the experimental groups. The bias resulting from repeated measurements was mitigated by testing each animal at the same time of day each day. Neurological assessment was performed by two investigators who had no knowledge of the experimental groups, and other phases of behavioral assessment and data analysis were performed blindly. The OD and image analyses were performed using ImageJ.

All statistical analysis and graphing were performed using the GraphPad Prism 9.0 software package (San Diego, CA).

## 3 Results

### 3.1 Olfactory performance testing

Animals were daily scored by EAE neurological scale, which reflects the severity of motor neurological symptoms (Figure 2B). In most animals, the first discrete motor signs appeared between 7 dpi and 9 dpi and then progressed fast in the next few days. Accordingly, the disease progression up to 8 dpi was referred to as the premotor phase, while the subsequent phase was referred to as the motor phase of EAE. Each animal was sacrificed when it reached the neurological score of 2.5–3, which corresponds to paraplegia and/or quadriparesis. At the time of sacrifice, EAE animals lost no more than 5.0% of their initial body mass (Figure 2B).

In parallel, animals were tested in a buried food test, which measures the time required by animals to find buried food. Animals were tested 3 days before EAE induction to establish their individual baseline latency (s). Each animal’s performance in BFT from 0 dpi afterwards was expressed as relative to the baseline latency (fold change) and the average daily latency ± (SD) was plotted in Figure 2B. A transient decline in olfactory performance was observed at ~2 dpi, as evidenced by more than 3-fold increase in latency to find buried food compared to the baseline latency before immunization (Figure 2B). After a brief recovery, a second, more pronounced increase in relative latency occurred at ~8 dpi, just with the appearance of discrete signs of motor impairment. Thereafter, relative latency in BFT increased in parallel with EAE score and development of motor impairments (Figure 2B). A one-way ANOVA with repeated measurements and Dunnett’s *post-hoc* comparison of the data revealed a statistically significant increase in latency to detect buried food at 3 dpi (*t* = 2.043, *p* < 0.021) and 7 dpi (*t* = 3.097, *p* < 0.0069) and thereafter compared to −1 dpi (i.e., before immunization).

Next, we examined the ability of animals to discriminate between familiar and non-familiar social odors (ODT) by determining the discrimination index (DI) (Figures 2C,D). Discrimination index of ~70% at −1 dpi indicated strong animals’ preference to explore the compartment with non-familiar odor. One-way ANOVA [*F* (1, 95, 10, 74) = 9.452, *p* < 0.0044] and Dunnett’s *post-hoc* tests showed a significant decrease in the DI values at 3 dpi (16.0 ± 41.3, *p* < 0.0132) and 7 dpi (24.9 ± 29.8, *p* < 0.0314), which indicated an reduced ability to discriminate social odors due to impaired olfactory ability.

The open field test (OFT) was used to evaluate general locomotion and anxiety-related behavior in EAE rats at 7 dpi, which reflect animal’s capacity and willingness to move. Parameters reflecting general locomotor activity, including distance traveled, average, and maximal speed, did not vary at 7 dpi with respect to pre-immunized animals (Table 1). Significant changes were observed in the amount of time spent in central and peripheral areas of the chamber and tendency of animals to remain close to walls, which strongly implied anxiety-like behavior (Figures 2E–I). EAE animals displayed less number of entries in peripheral fields (Figure 2F, *t* = 8.259, df = 9, *p* < 0.0001), increased time spent in peripheral fields (Figure 2G, *W* = 55, *p* = 0.002), and increased number of immobile episodes (Figure 2H, *p* < 0.01) and total immobile time spent in peripheral fields (Figure 2I, *W* = 45, *p* = 0.0039) compared to their performance at −1 dpi. All parameters obtained in OFT are summarized in Supplementary Table S4.

**Table 1:** Motor parameters from OFT.

Number of entries in central fields	1.4 ± 1.4	0.7 ± 1.1
	(*t* = 0.9575, df = 9, *p* = 0.363)	
Time spent in central fields (s)	13.0 ± 22.4	3.0 ± 5.0
	(*W* = −23, *p* = 0.275)	
Distance traveled in central fields (m)	0.7 ± 0.7	0.3 ± 0.6
	(*W* = −21, *p* = 0.322)	
Average speed in central fields (m/s)	0.05 ± 0.06	0.03 ± 0.05
	(*t* = 0.6359, df = 9, *p* = 0.541)	
Maximal speed in central fields (m/s)	0.20 ± 0.18	0.13 ± 0.17
	(*t* = 0.6482, df = 9, *p* = 0.533)	
Mobile time in central fields (s)	13.0 ± 22.3	3.0 ± 5.0
	(*t* = 1.277, df = 9, *p* = 0.233)	
Time spent in peripheral fields (s)	457 ± 44	583 ± 34
	(*W* = 55, *p* = 0.002*)	
Distance traveled in peripheral fields (m)	16.6 ± 4.6	12.6 ± 5.1
	(*t* = 2.615, df = 9, *p* = 0.028*)	
Average speed in peripheral fields (m/s)	0.04 ± 0.01	0.02 ± 0.01
	(*t* = 4.118, df = 9, *p* = 0.0026*)	
Maximal speed in peripheral fields (m/s)	0.52 ± 0.11	0.48 ± 0.11
	(*t* = 1.113, df = 9, *p* = 0.2944)	
Mobile time spent in peripheral fields (s)	450 ± 51	475 ± 74
	(*t* = 1.149, df = 9, *p* = 0.2803)	

Novel object recognition test (NORT) was conducted to assess cognitive performance in EAE animals (Figure 2J). Intact animals have natural tendency to explore novel unfamiliar objects. At −1 dpi, animals spent greater amount of time with the novel object, while at 7 dpi, they did not show a preference for the novel object, demonstrated by significantly lower decrease in recognition index (RI) at 7 dpi compared to −1 dpi (*t* = 2.227, df = 9, *p* = 0.0488). Parameters obtained in NORT are summarized in Supplementary Table S5.

### 3.2 Assessment of neuroinflammation in OB

**Figure 3:**
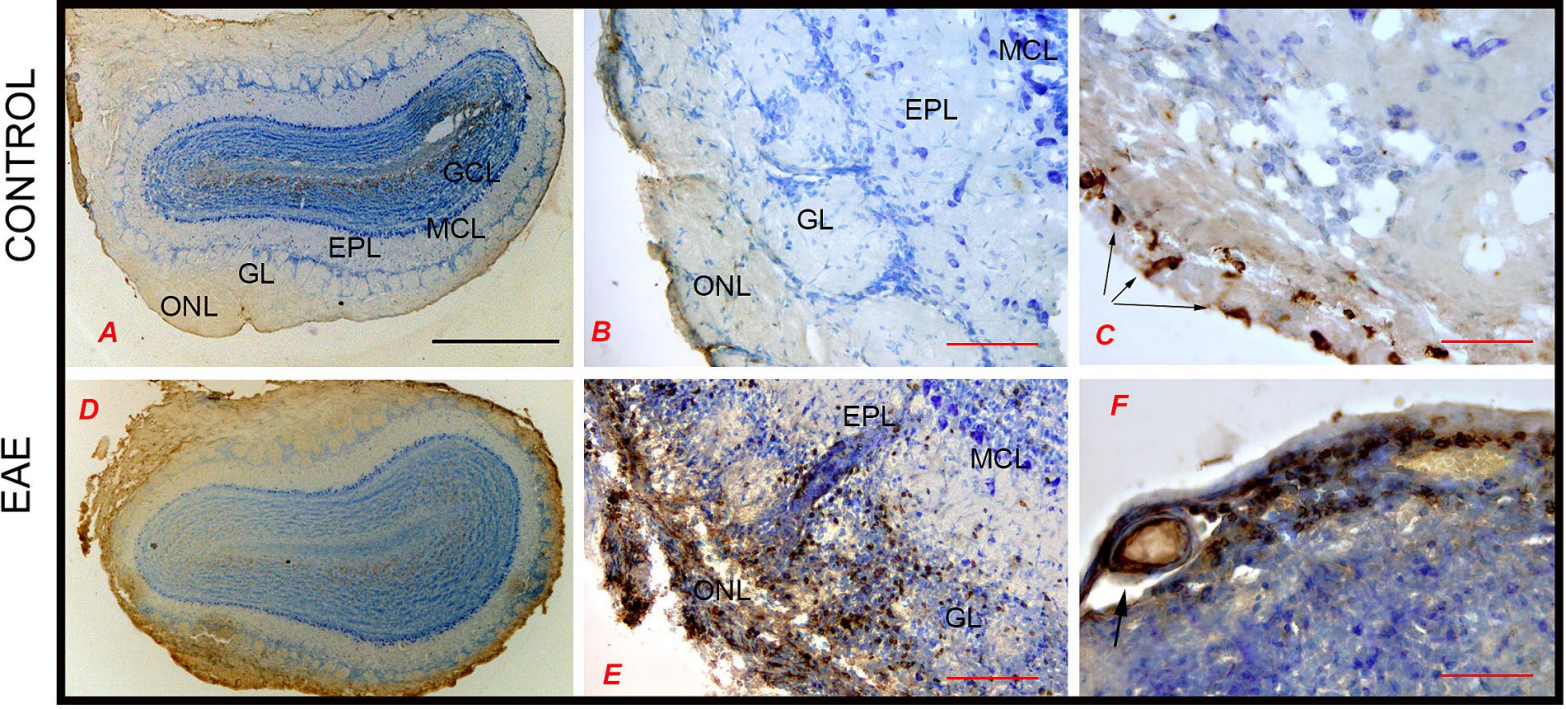
CD4-directed immunohistochemical labeling of the olfactory bulb. **(A–C)** In control sections, CD4^+^ cells were not seen within OB tissue; only a few CD4^+^ T cells were restricted to the meningeal surface (arrows). **(D,E)** In EAE sections, numerous CD4^+^ T cells infiltrated the ONL from the meningeal side and reached the GL and EPL. **(F)** CD4^+^ cells around the blood vessels. Scale bar: **(A,D)** 1,000 μm; **(B,E)** 200 μm; **(C,F)** 100 μm. GCL, granule cell layer; GL, glomerular layer; EPL, external plexiform layer; MCL, mitral cell layer; ONL, olfactory nerve layer.

The observed decrease in olfactory performance and odor-related behavior in EAE prompted us to investigate the histopathological status of OB. It has been previously postulated that neuroinflammation decreased plasticity and renewal of interneurons in the OB are the underlying mechanisms of olfactory dysfunction in EAE (Tepavčević et al., 2011). The presence of infiltrated CD4^+^ cells in OB was investigated by CD4-directed immunohistochemistry (Figure 3). CD4^+^ cells were not present in healthy OB tissue; only a few CD4^+^ cells were restricted to the meningeal surface in the control OB (Figures 3A–C). In EAE sections, numerous CD4^+^ cells infiltrated the OB in the EAE (Figures 3D–F). CD4^+^ cells were accumulated along the axons of the olfactory nerve in the ONL, GL, and EPL (Figure 3E) and around the blood vessels (Figure 3F). Since massive infiltrates were seen on the EAE sections, the images are a qualitative confirmation of a disturbed blood–brain barrier and infiltration with peripheral cells.

**Figure 4:**
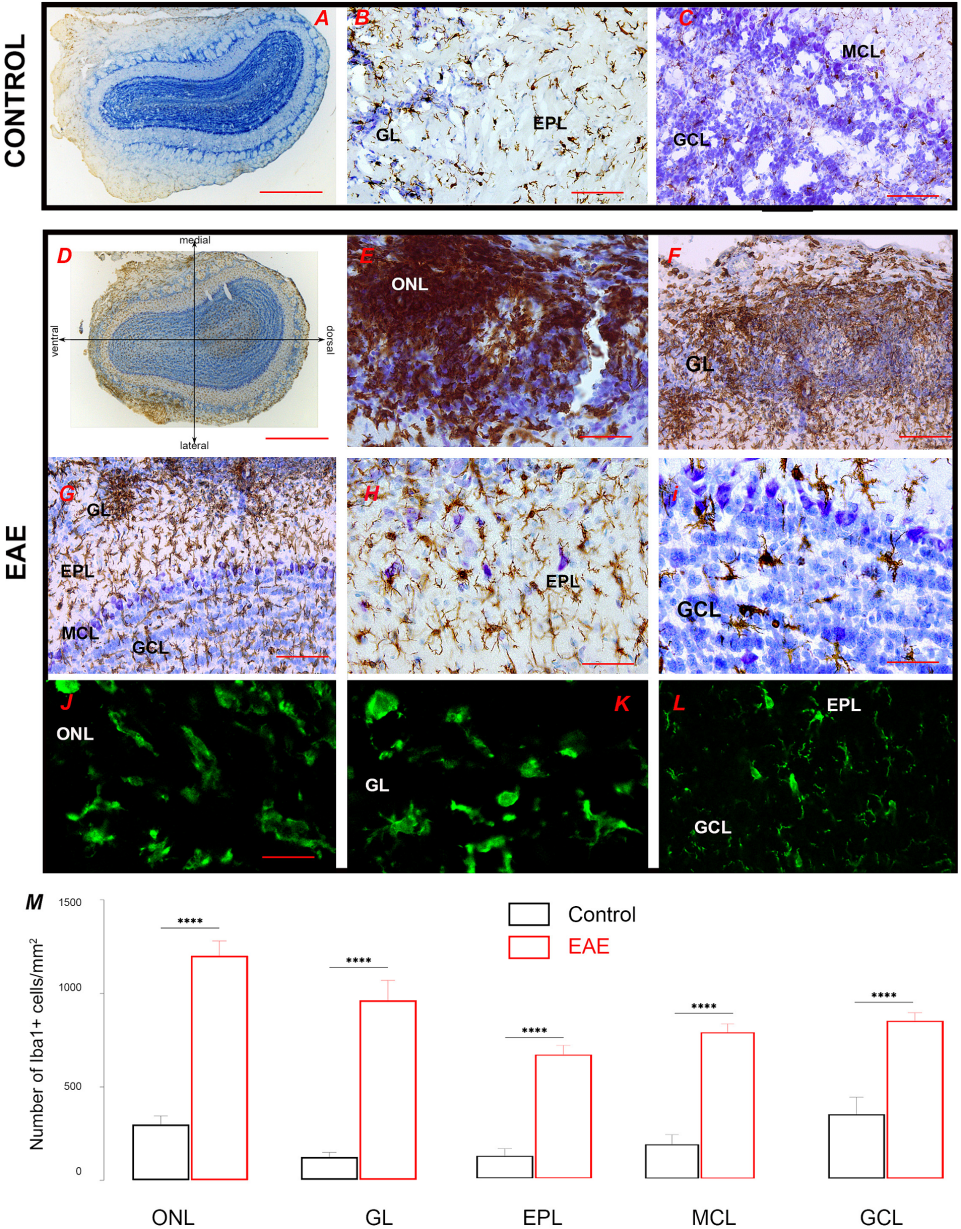
Iba1-directed immunohistochemical labeling of the olfactory bulb. **(A–C)** Iba1-*ir* labeling of the control section shows quiescent ramified microglia in all OB layers. **(D)** Iba1-*ir* in the EAE section relative to lateral-to-medial OB axis; numerous Iba1^+^ cells were seen in the ONL on the lateral OB side. **(E,F)** Clusters of Iba1-*ir* cells belonging to infiltrated macrophages and highly reactive ovoid microglia in the ONL and GL. **(G)** Ramified microglia in the deeper OB layers. Microglia with a slightly reactive phenotype but organized in a non-overlapping manner in **(H)** EPL and **(I)** MCL and GCL. Confocal Iba1 immunofluorescence confirms the presence of ovoid Iba1-*ir* cells in **(J)** ONL and **(K)** GL, and typical ramified microglia with preserved domain organization in **(L)** EPL and GCL. **(M)** Graphs showing the number of reactive Iba1^+^ cells in distinct cell layers in EAE sections. Statistical significance: ****p < 0.0001. Scale bar: 1000 μm in **(A,D)**; 100 μm in **(B,C,E,F,G)**; 50 μm in **(H,I)**; 20 μm in **(J–L)**. GCL, granule cell layer; GL, glomerular layer; EPL, external plexiform layer; MCL, mitral cell layer; ONL, olfactory nerve layer.

Iba1 immunohistochemistry was performed to detect infiltrated monocytes/macrophages and resident microglia cells. In control OB, Iba1-*ir* labeled quiescent microglial cells, with small cell bodies and thin radial processes, which were evenly distributed throughout the OB (Figures 4A–C). In EAE, Iba1-*ir* was much more prominent throughout the OB, with distinct patterns of Iba1-*ir* in the superficial and deeper OB layers (Figure 4D). Specifically, in the ONL, Iba1-*ir* labeled numerous monocytes/macrophages and highly reactive ameboid microglia (Figures 4D,E), which were more abundant on the lateral side than on the medial side of the OB. In the GL, clusters of highly reactive microglial cells lost their domain organization (Figure 4F). However, in the EPL and GCL, especially in the parts that did not show infiltrates (Figures 4G–I), microglial cells showed slightly reactive morphology but preserved their domain organization (Figures 4G–I). Iba1-directed immunofluorescence confirmed differences in microglial shape/reactivity from the superficial to inner OB layers (Figures 4J–L). Quantification of Iba1-ir signal intensity shows the spatial gradient of Iba1^+^ cell density from the ONL to the innermost OB layer (Figure 4M), i.e., the density of Iba1^+^ cells observed gradually decreases from the ONL to the center of the OB.

**Figure 5:**
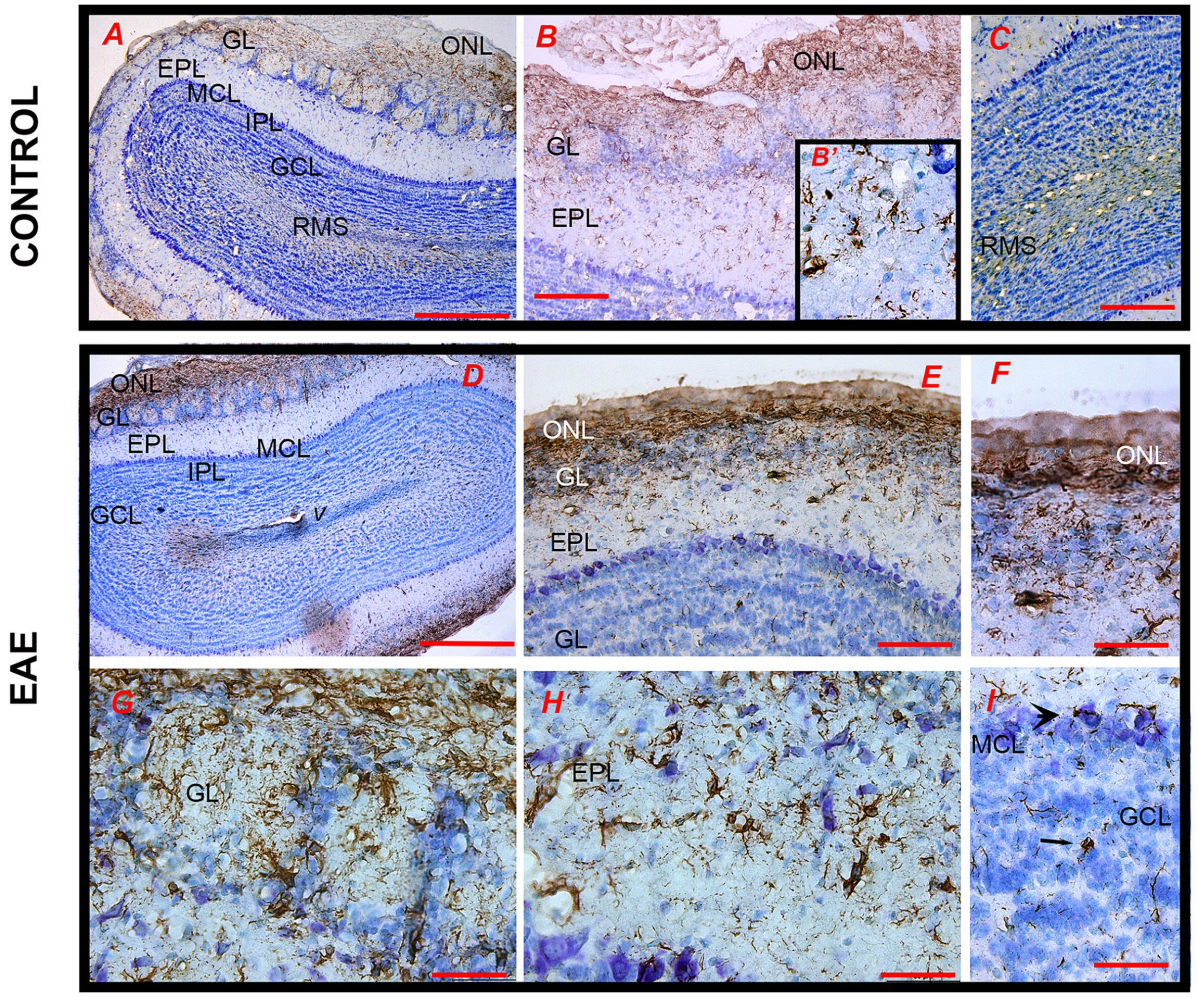
GFAP-directed immunohistochemical labeling of the olfactory bulb. **(A)** The pattern of GFAP-*ir* in the control section at low magnification. **(B)** GFAP-*ir* mainly marks astrocytes in the ONL, GL (magnified in **B′**), and **(C)** RMS. **(D,E)** GFAP-*ir* in the EAE section shows slightly hypertrophied astrocytes in **(F)** ONL, **(G)** GL, and **(H)** EPL with non-overlapping distribution, and a less intense GFAP-*ir* in the RMS. GCL, granule cell layer; GL, glomerular layer; EPL, external plexiform layer; IPL, internal plexiform layer; MCL, mitral cell layer; ONL, olfactory nerve layer; RMS—rostral migratory stream; V—tip of the lateral ventricle. Scale bar: 500 μm in **(A,D)**; 200 μm in **(B,C,E)** and 50 μm in **(B′,F–I)**.

Astrocyte reactivity in the inflamed OB was analyzed by GFAP immunohistochemistry (Figure 5). In the control OB, GFAP-*ir* labeled a network of OEC processes in the ONL and typical astrocytes in successive OB layers (Figure 5B). In the center of the OB, GFAP-*ir* moderately labeled glial tubes bordering the RMS (Figure 5C). In EAE, astrocytes showed signs of hypertrophy, reflected as moderately increased overall protein abundance of GFAP (Supplementary Figure S1), although the cells retained the non-overlapping arrangement (Figure 5D). In the ONL, GFAP-*ir* labeled ticked processes of OEC and slightly activated astrocytes (Figures 5E,F), while in the successive layers, astrocyte hypertrophy gradually decreased (Figures 5G,H), so that astrocytes in the GL showed a similar morphology to those of controls (Figure 5I, arrow).

As a consequence of the massive infiltration of immune cells and a strong activation of glial cells, we expected further signs of neuroinflammation. Quantitative RT-PCR was used to assess expression of the proinflammatory mediators of glial activation in EAE. Student’s *t*-test showed several-fold induction of genes encoding for TNF-α, IL-1β, IL-6, complement component C3, and lipocalin-2 (Lcn) (Table 1).

### 3.3 Assessment of neuronal plasticity in OB

**Figure 6:**
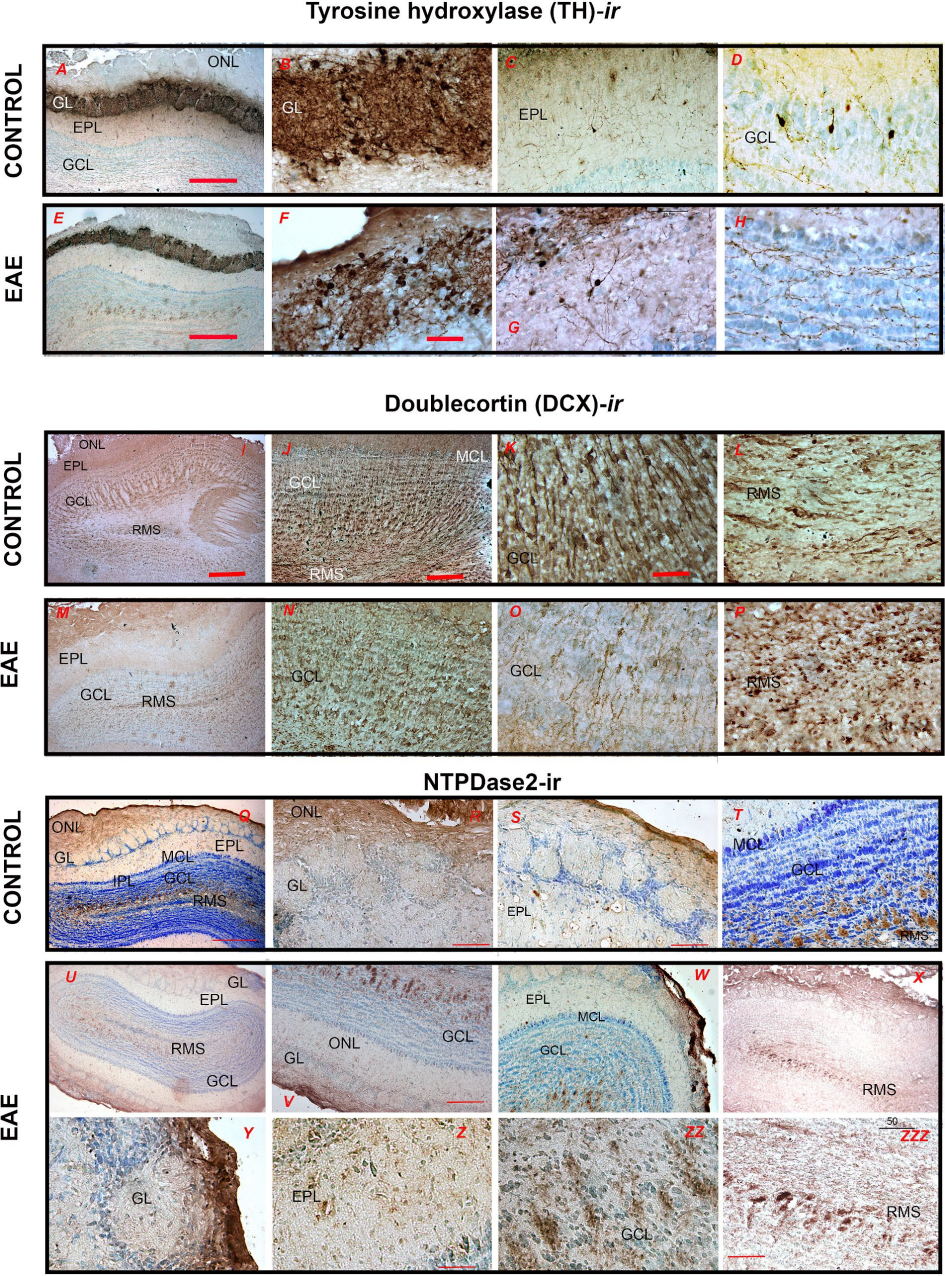
Immunohistochemical labeling of OB cells derived from postnatal neurogenesis. **(A–D)** Tyrosine hydroxylase (TH)-immunohistochemistry in control and **(E–H)** EAE sections. **(I–L)** Doublecortin (DCX)-directed immunohistochemistry in control and **(M–P)** EAE sections. **(Q–T)** NTPDase2-directed immunohistochemistry in control and (U–X) EAE sections. GCL, granule cell layer; GL, glomerular layer; EPL, external plexiform layer; IPL, internal plexiform layer; MCL, mitral cell layer; ONL, olfactory nerve layer; RMS—rostral migratory stream. Scale bar: 500 μm in **(I,M,Q,U)**; 200 μm in **(A,E,V,W,X)**; 100 μm in **(C,D,G,H,L,O,P,Q,R,S,Y,Z)**; 50 μm in **(K,N,ZZ,ZZZ)**.

The plasticity of neuronal networks in OB was analyzed by immunohistochemistry directed to tyrosine hydroxylase (TH), doublecortin (DCX), and NTPDase2. Tyrosine hydroxylase was used as a marker for dopaminergic neurons. In the control OB, very strong TH-*ir* labeled small to medium cell bodies in the GL (Figure 6A) and the dense network of their neurites extending between the glomeruli (Figure 6B). TH-*ir* also stained sparse medium-sized neurons in EPL (Figure 6C) and GCL (Figure 6D). Although the overall intensity of TH-*ir* apparently decreased in EAE (Figures 6E–H; Supplementary Figure S3A), particularly in periglomerular neurites (Figure 6F), the number of TH-ir cell bodies increased in EAE in respect to control (*p* < 0.0001) (Supplementary Figure S3B).

**Figure 7:**
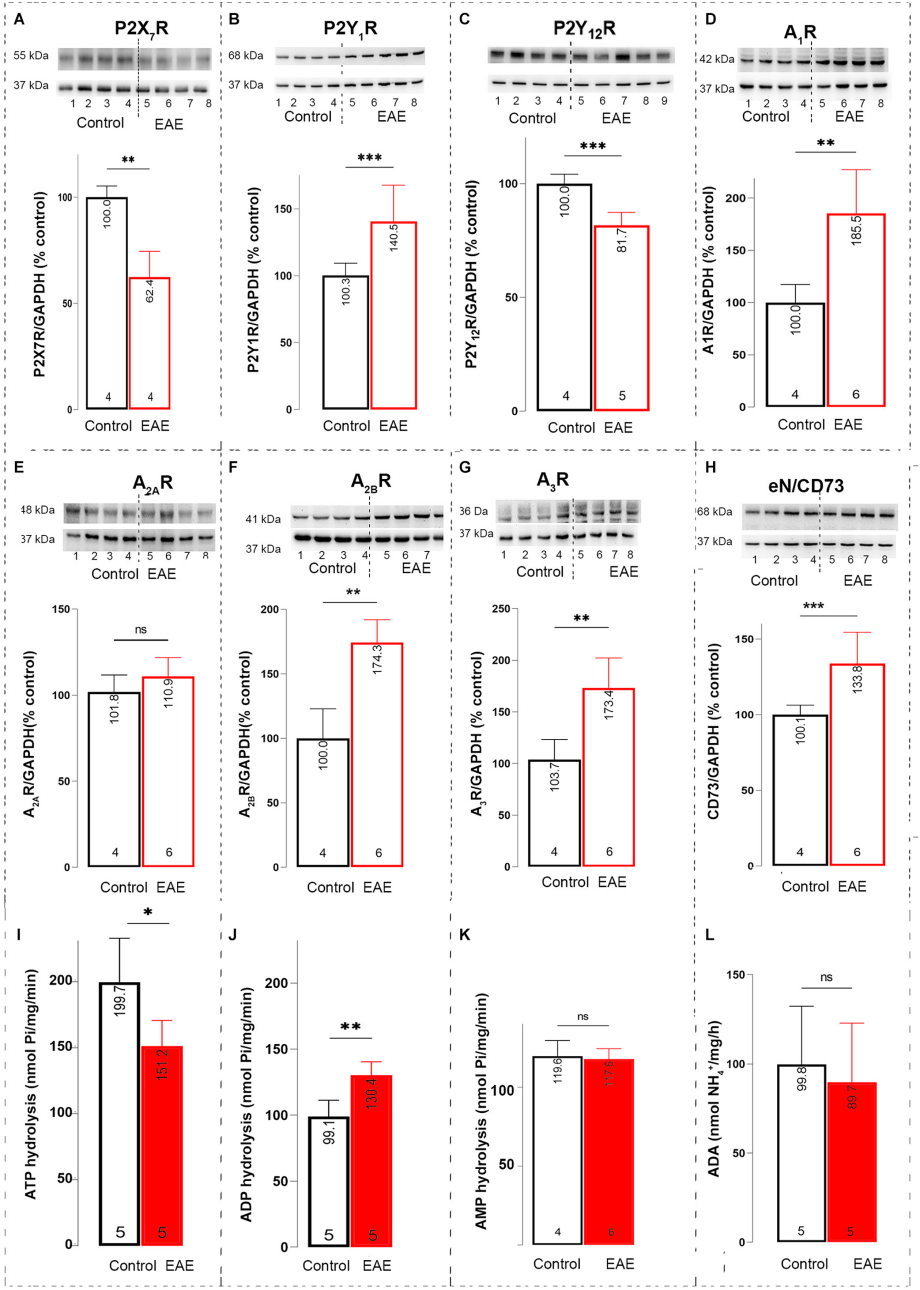
Purinome expression in OB. Representative immunoblot membranes and graphs showing the relative protein abundance of **(A)** P2X_7_R, **(B)** P2Y_1_R, **(C)** P2Y_12_R, **(D)** A_1_R, **(E)** A_2A_R, **(F)** A_2B_R, **(G)** A_3_R, and **(H)** eN/CD73 in olfactory bulb P2 fractions isolated from control animals (*black bar*) and EAE animals (*red bar*). Data shown in the graphs represent the relative target protein/GAPDH abundance compared to control, expressed as mean ± SD, obtained by blotting four P2 samples/experimental group in two independent replicates. Rates of **(I)** ATP hydrolysis, **(J)** ADP hydrolysis, and **(K)** AMP hydrolysis in olfactory bulb P2 fractions isolated from control (*black bar*) and EAE animals (*red bar*). Data are expressed as mean nucleotide hydrolysis (nmol Pi/mg/min) ± SD determined in four P2 samples/experimental group run in triplicate and repeated two times. **(L)** Rate of adenosine deamination in olfactory bulb P2 fractions isolated from control (*black bar*) and EAE animals (*red bar*). Data are expressed as mean activity (nmol NH_4_^+^/mg/h) ± SD determined in four P2 samples/experimental group run in triplicate and repeated two times. Significance shown inside the graphs: *p < 0.05; **p < 0.01; ***p < 0.001.

Doublecortin (DCX) was used as a marker for actively dividing neuroblasts. In the control OB, DCX-*ir* labeled migrating type A cells in the RMS (Figures 7I–L), which are responsible for the continual replacement of granular and periglomerular interneurons. In EAE, the density of DCX-*ir* cells decreased significantly both in the RMS (Figure 7M) and in GCL and GL (Figures 7N,O). In addition to the reduced number, the DCX-*ir* cells in the GCL also changed their spatial arrangement (Figure 7N), while their processes showed conspicuous signs of atrophy (Figure 7O). Furthermore, DCX-*ir* cells in the RMS lost their bipolar shape and shortened processes (Figure 7P), suggesting a reduced replacement of GL and GCL interneurons and overall impaired plasticity in EAE (Table 2).

**Table 2:** Target gene/GAPDH-mRNA abundance.

Target gene	Control	EAE (2^−ΔCt^; % of control; statistical difference)
↑ *Entpd1*	0.01984 ± 0.00208	0.03253 ± 0.00813 (164.0 ± 11.3%, *t* = 2.52, df = 8, *p* = 0.0356)
↓ *Entpd2*	0.03414 ± 0.00107	0.02169 ± 0.0038 (63.5 ± 23.0%, *t* = 4.87, df = 8, *p* = 0.0012)
↑ *Nt5e*	0.01166 ± 0.00200	0.01754 ± 0.00052* (150.4 ± 24.6%, *t* = 13.35, df = 8, *p* < 0.0001)
*Ada*	0.00237 ± 0.00032	0.00212 ± 0.00057 (*t* = 0.95, df = 8, *p* = 0.3571)
*Ent1*	0.00225 ± 0.00039	0.00225 ± 0.00060 (*t* = 0.13, df = 8, *p* = 0.9896)
*Ent2*	0.00367 ± 0.00032	0.00259 ± 0.00048 (*t* = 0.13, df = 8, *p* = 0.9896)
↑ *Adora1*	0.01563 ± 0.00065	0.01986 ± 0.00513* (127.1 ± 9.6%, *t* = 2.63, df = 8, *p* = 0.0206)
*Adora 2a*	0.00374 ± 0.00019	0.00333 ± 0.00078 (*t* = 0.92, df = 8, *p* = 0.3832)
*Adora2b*	0.00522 ± 0.00068	0.00412 ± 0.00071 (*t* = 1.90, df = 8, *p* = 0.0937)
↑ *Adora3*	0.00900 ± 0.00128	0.02558 ± 0.0038* (284.2 ± 72.33%, *t* = 6.375, df = 8, *p* = 0.0002)
*P2xr2*	0.00037 ± 0.00014	0.00030 ± 0.00061 (*t* = 1.61, df = 8, *p* = 0.1298)
↑ *P2xr4*	0.02456 ± 0.00433	0.04073 ± 0.00829* (165.8 ± 12.3, *t* = 5.59, df = 8, *p* < 0.0001)
*P2xr7*	0.00373 ± 0.00060	0.00375 ± 0.00107 (*t* = 0.40, df = 8, *p* = 0.6929)
*P2ry1*	0.00535 ± 0.00062	0.00484 ± 0.00096 (*t* = 2.04, df = 8, *p* = 0.059)
↑ *P2ry12*	0.00869 ± 0.00162	0.01210 ± 0.00159* (139 ± 1.9%, *t* = 4.24, df = 8, *p* = 0.0007)
↑ *Tnfa*	0.00055 ± 0.00005	0.00258 ± 0.00147* (469.1 ± 185.0%, *t* = 3.78, df = 8, *p* = 0.0054)
↑*Il1b*	0.00053 ± 0.00015	0.00439 ± 0.00236* (828.3 ± 298.4%, *t* = 3.19, df = 8, *p* = 0.0127)
↑ *Il6*	0.00004 ± 0.00010	0.00024 ± 0.00005* (600.0 ± 35.7%, *t* = 3.46, df = 8, *p* = 0.0085)
↑*Lcn2*	0.00007 ± 0.00002	0.00117 ± 0.00050* (1,671 ± 140.4%, *t* = 3.40, df = 8, *p* = 0.0093)
↑*C3*	0.02241 ± 0.00369	0.12017 ± 0.02740* (536.2 ± 180.5%, *t* = 5.50, df = 8, *p* = 0.0006)

NTPDase2 immunohistochemistry was used to label type B cells, which are astrocyte-like type B cells that form glial tubes as a scaffold for the migration of type A neuroblasts in the RMS. In control OB, strong NTPDase2-*ir* labeled OEC cell bodies and processes in the ONL (Figures 7Q,R) and RMS (Figure 7T). In EAE, NTPDase2-*ir* was slightly less intense overall (Figure 7U), although the pattern of NTPDase2-*ir* in the ONL (Figure 7V) was comparable to that in the control OB. In the EPL, however, only slight somatic labeling was observed (Figure 7Z). The difference in NTPDase2-*ir* was most pronounced in the RMS, where NTPDase2-*ir* cells were less numerous and less conspicuous (Figure 7ZZZ).

### 3.4 Purinergic signaling in OB

We first determined the purinome expression in OB under control conditions and in EAE (Table 1). Significant changes in gene expression in EAE compared to control were observed for *P2rx4*, *P2ry12*, *Adora1*, *Adora3*, *Entpd1*, *Entpd2*, and *Nt5e*. Consistent changes at protein level were observed for eN/CD73 (Figure 7H, *t* = 4.40, df = 6, *p* = 0.0004), A_1_R (Figure 7D, *t* = 6.80, df = 6, *p* < 0.01), A_3_R (Figure 7G, *t* = 3.98, df = 6, *p* = 0.0073), and P2Y_12_R (Figure 7C, *t* = 5.42, df = 6, *p* = 0.001). The abundance of P2X_7_R (Figure 7A, *t* = 5.70, df = 6, *p* = 0.0013), P2Y_1_R (Figure 7B, *t* = 4.47, df = 6, *p* = 0.0002), and A_2B_R (Figure 7F, *t* = 5.15, df = 6, *p* = 0.0021) significantly increased at the protein level only. Representative PVDF membranes are presented in Supplementary Figure S2.

The activities of ectonucleotidase enzymes were further determined in OB. The rate of ATP hydrolysis (Figure 7I), indicative of both NTPDase2 and NTPDase1, significantly decreased in EAE (151.2 ± 19.4 nmol Pi/mg/min, *t* = 2.75, df = 7, *p* = 0.0285) compared to control (199.7 ± 33.33 nmol Pi/mg/min). On the other hand, the rate of ADP hydrolysis (Figure 7J), indicative of NTPDase1, increased in EAE (130.4 ± 9.916 nmol Pi/mg/min, *t* = 4.27, df = 7, *p* = 0.0037) compared to control (99.06 ± 12.2 nmol Pi/mg/min). The rates of AMP hydrolysis (Figure 7K, *t* = 0.56, df = 7, *p* = 0.5643) and adenosine deamination (Figure 7L, *t* = 0.66, df = 7, *p* = 0.4569) catalyzed by eN/CD73 and ADA, respectively, remained steady in EAE.

### 3.5 Tissue and cellular distribution of eN/CD73 and P1 receptors

**Figure 8:**
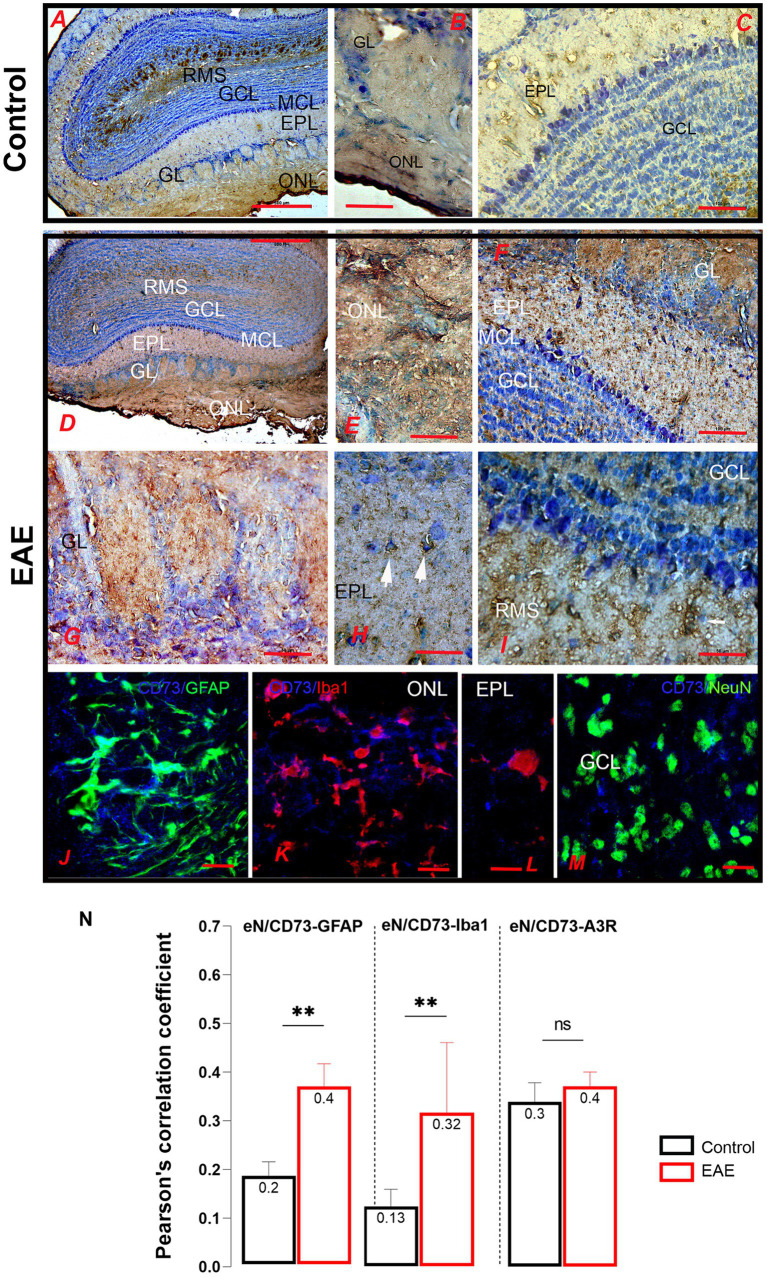
eN/CD73-directed immunohistochemical labeling of the olfactory bulb. **(A–C)** The pattern of eN/CD73-*ir* labeling in the ONL, GL, and EPL implies dominant astrocyte localization. **(D–I)** eN/CD73-*ir* in EAE sections. **(E)** Intense labeling of OEC cells and **(F)** astrocytes in the GL, EPL, and GCL astrocytes. **(H)** Sporadic labeling of tufted cells (arrows). **(J)** Double immunofluorescence directed to eN/CD73 (blue) and GFAP (green) showed astrocytic localization. **(K–L)** Double immunofluorescence directed to eN/CD73 (blue) and Iba1 (red) demonstrates induction of eN/CD73 in reactive microglia. **(M)** Double immunofluorescence directed to eN/CD73 (blue) and NeuN (green), indicating a possible presynaptic localization in mitral and tufted cell dendrites. **(N)** Pearson’s correlation coefficient (PCC) shows a degree of overlap between the fluorescence signals. GCL, granule cell layer; GL, glomerular layer; EPL, external plexiform layer; IPL, internal plexiform layer; MCL, mitral cell layer; ONL, olfactory nerve layer, RMS—rostral migratory stream. Scale bar: 500 μm in **(A,D)**; 100 μm in **(C,F)**; 50 μm in **(B,E,G,H,I)**; 20 μm in **(J–L)**. Significance is shown within the graph: **p < 0.01.

In the course of the research, we focused on the components of adenosine signaling whose expression is altered in EAE. Cell distribution was analyzed by immunohistochemistry and confocal immunofluorescence. In the control OB, strong eN/CD73-*ir* labeled the ONL parenchyma (Figures 8A,B), whereas the pattern of eN/CD73-*ir* in the GL (Figure 8B), EPL, and GCL (Figure 8C) indicated dominant astrocyte localization. Intense eN/CD73-*ir* labeled the astrocyte-like cells lining the glial tubes in the RMS (Figure 8A). The overall intensity of eN/CD73-*ir* fluorescence increased in EAE compared to control (Figure 8D). The intensity of parenchymal labeling increased in the ONL, although it was difficult to detect individual cell bodies and processes of OECs (Figure 8E). The eN/CD73-*ir* was more prominent in the GL, EPL, and GCL astrocytes (Figure 8F). At higher magnification, sporadic perisomatic labeling of tufted cells was also observed in the EPL (Figure 8H, arrows), whereas the immunoreaction was less pronounced in the RMS compared to the control (Figure 8I). Double immunofluorescence confirmed a significant overlap between eN/CD73-*ir* and GFAP-*ir* (Figure 8J), confirming an increase in astrocytic expression of eN/CD73. However, a substantial portion of eN/CD73-*ir* also overlapped with Iba1-*ir*, indicating expression in infiltrated immune cells and/or reactive microglia (Figures 8K,L). A distinct eN/CD73-*ir* pattern was observed around the bodies and proximal dendrites of granule cells, indicating a possible presynaptic localization in glutamatergic dendritic inputs of mitral and tufted cells (Figure 8M). Pearson’s correlation coefficient (PCC) was determined to assess the degree of overlap between two fluorescent signals (Figure 8N). A 2-fold increase in PCC value for the overlap of eN/CD73-GFAP in EAE compared to control (*t* = 3.33, df = 37, *p* = 0.0059) and the 3-fold increase in PCC value for the overlap of eN/CD73 and Iba1 (*t* = 3.19, df = 36, *p* = 0.0096) confirmed that the increase in eN/CD73 expression occurred in both reactive astrocytes and microglia.

**Figure 9:**
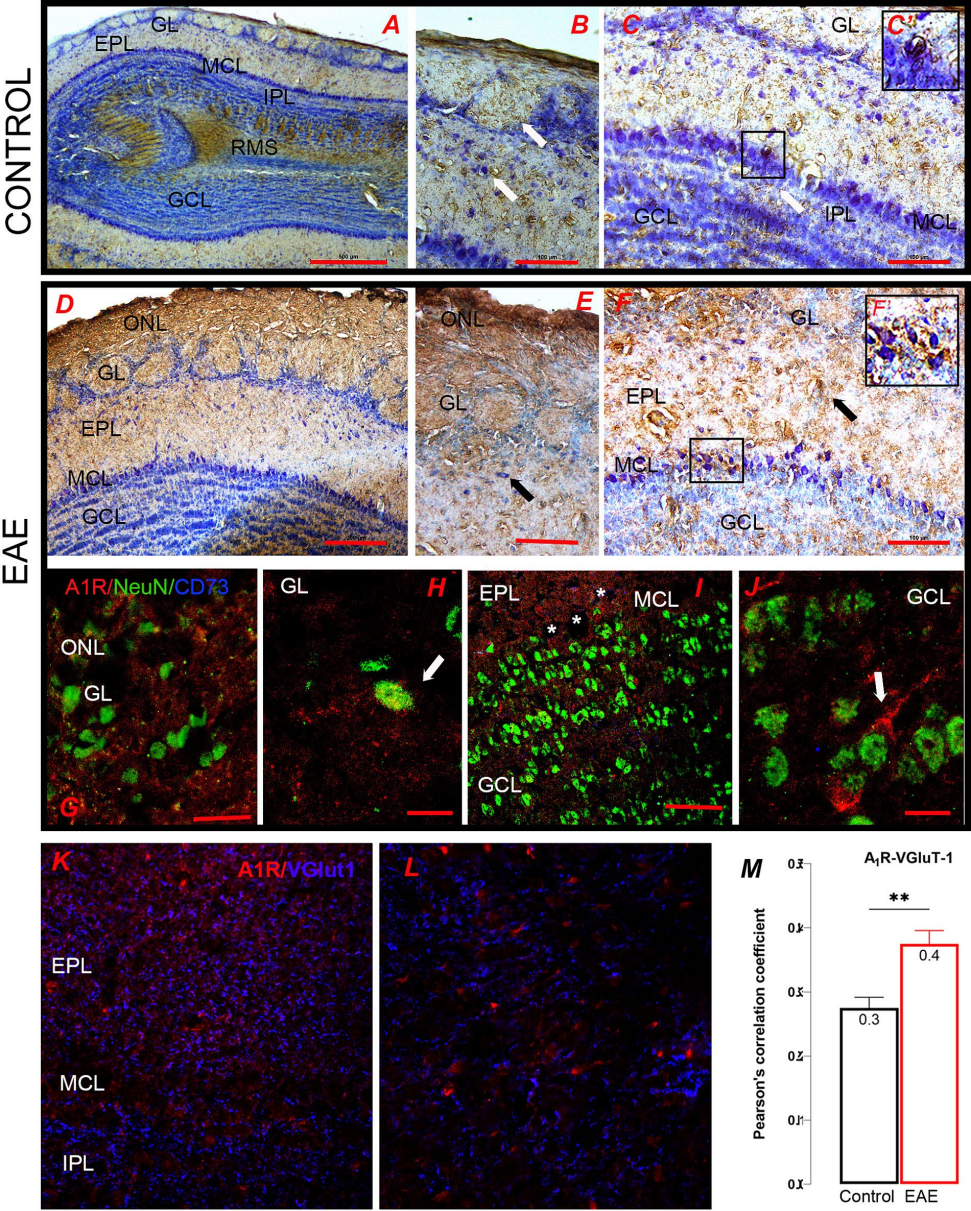
A_1_R-directed immunohistochemical labeling of the olfactory bulb. **(A–C)** Control sections were immunostained for A_1_R. **(B)** The somatic A_1_R-*ir* was seen in the cell bodies of periglomerular neurons and **(C)** tufted cells and mitral cells. The frame enlarged in **C′** shows A_1_R-*ir* mitral cells. **(D)** A_1_R-*ir* in the EAE section shows increased ir in the ONL, **(E)** EPL and MCL, and **(F)** GCL. **(F′)** High-power magnification of the area enclosed by rectangle in **(F)**. **(G–H)** Double immunofluorescence directed to A_1_R (*red*) and NeuN (*green*) shows **(G,H)** the somatic localization of A_1_R in perglomerular neurons and **(I–J)** in granule cells. Asterisks in **(I)** denote A_1_R-*ir* mitral cells which do not express NeuN. **(K–L)** Double immunofluorescence directed to A_1_R (*red*) and Vglut1 (*blue*), which show prominent overlap of the signals in **(K)** EPL and **(L)** IPL. **(M)** Pearson correlation coefficient (PCC) reflecting an increase in A_1_R/VGlut1 overlap in EAE in comparison to control. GCL, granule cell layer; GL, glomerular layer; EPL, external plexiform layer; IPL, internal plexiform layer; MCL, mitral cell layer; ONL, olfactory nerve layer, RMS—rostral migratory stream. Scale bar: 500 μm in **(A)**; 200 μm in **(D)**; 100 μm in **(B,C,E,F)**; 50 μm in **(C′,F′,G,I,K,L)**; 20 μm in **(H,J)**. Significance within the graph: *p < 0.01.

As for adenosine receptors, significant basal expression of A_1_R was detected in OB, especially in GL, EPL, and MCL (Figure 9A). At higher magnification, A_1_R-*ir* was localized in the cell bodies of periglomerular neurons in the GL, tufted cells (Figure 9B), and mitral cells (Figure 9C). The intensity of A_1_R-*ir* increased throughout the OB in EAE (Figure 9D). The more intense A_1_R-*ir* was observed in the ONL parenchyma (Figure 9D) and in the cell bodies of tufted, mitral, and granule neurons (Figures 9E,F). The increase in the somatic localization of A_1_R was confirmed by double immunofluorescence directed against A_1_R and NeuN (*green*) (Figures 9G–J). The increase in A_1_R fluorescence intensity was also observed in mitral cells, which are among the rare neurons that do not express NeuN and therefore remained unlabeled in the microscopic sections (Figure 9I, *asterisks*). Nevertheless, a significant part of extrasomatic A_1_R fluorescence was observed in the EPL (Figure 9I), which harbors dendro-dendritic and dendrosomatic synapses between mitral/tufted cells and granule cells (Figure 9J, *arrow*). Alteration in presynaptic A_1_R in EAE was examined with double immunofluorescence directed to A_1_R and VGlut1, the latter being used as a marker for glutamatergic synapses. VGlut1 and A_1_R immunofluorescence signals were closely localized in the EPL and IPL (Figures 9K,L), whereas a significant increase in PCC value for the overlap of A_1_R-VGlut1 signals in EAE indicated an increase in synaptic expression of A_1_R (Figure 9M, *t* = 3.42, df = 42, *p* = 0.0019).

**Figure 10:**
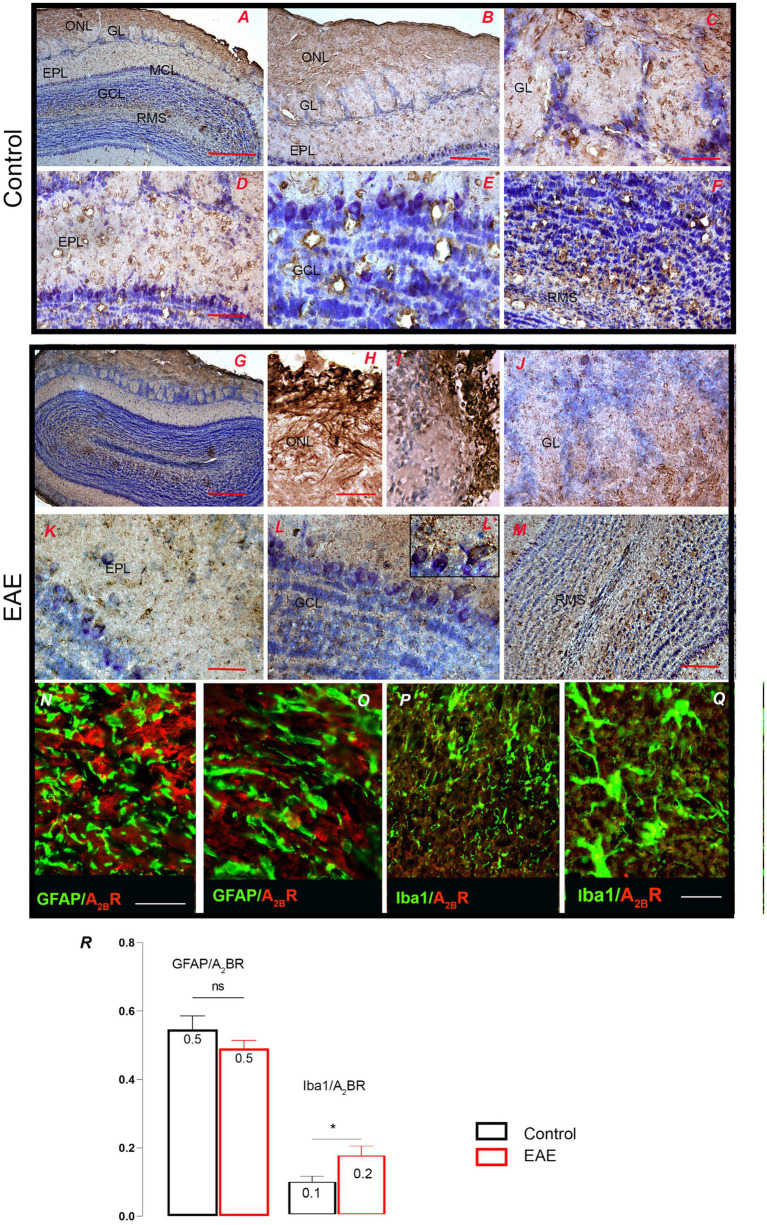
A_2B_R-directed immunohistochemical labeling of the olfactory bulb. **(A,B)** The pattern of A_2B_R-*ir* in control OB shows dominant astrocytic localization in **(C)** GL, **(D)** EPL, and **(E)** GCL and distinct labeling in **(F)** RMS. **(G)** Low magnification micrograph shows A_2B_R-*ir* in the EAE section, with increased labeling in **(H)** ONL and **(I)** infiltrated cells and similar labeling in **(J)** GL and **(K)** EPL. **(L)** The high-power micrograph shows conspicuous A_2B_R labeling of mitral cells (magnified in **L′**) and **(M)** conspicuous labeling in RMS. **(N,O)** Double immunofluorescence directed to A_2B_R (*red*) and GFAP (*green*) confirms the overlap of the two signals. **(P,Q)** Double immunofluorescence directed to A_2B_R (*red*) and Iba1 (*green*) shows the presence of A_2B_R in reactive microglia. **(R)** PCC values for A_2B_R/GFAP and A_2B_R/Iba1 signal overlap in the control (*black bars*) and EAE (*red bars*). GCL, granule cell layer; GL, glomerular layer; EPL, external plexiform layer; IPL, internal plexiform layer; MCL, mitral cell layer; ONL, olfactory nerve layer; RMS—rostral migratory stream. Scale bar: 500 μm in **(A,G)**; 200 μm in **(B,J,M)**; 100 μm in **(D,F)**; 50 μm in **(C,E,H,I,K,L,N,P)**; 20 μm in **(O,Q)**. Significance within the graph: *p < 0.05.

A_2A_R immunohistochemistry in OB showed similar patterns in control and EAE (Supplementary Figure S4). The pattern of A_2B_R-*ir* largely overlapped with the pattern of GFAP-*ir* in control OB (Figures 10A,B), indicating a dominant astrocytic localization in GL (Figure 10C), EPL (Figure 10D), and GCL (Figure 10E). A pronounced A_2B_R-*ir* was observed in the RMS (Figure 10F). In EAE, a slightly pronounced A_2B_R-*ir* was observed in the ONL (Figure 10G), where it labeled OECs (Figure 10H) and infiltrated cells (Figure 10I). The pattern of A_2B_R-*ir* in GL and EPL was similar to the control (Figures 10J–L). Slightly more pronounced A_2B_R-*ir* was observed in the mitral cells (Figure 10L) and in the RMS (Figure 10M). Double immunofluorescence directed against A_2B_R and GFAP confirmed a clear overlap between the two fluorescent signals (Figures 10N,O). The same was true for the overlap between A_2B_R and Iba1 (Figures 10P,Q) in EAE. The high PCC value for the A_2B_R-GFAP signal overlap and the low PCC value for the A_2B_R/Iba1 signal overlap confirm the dominant astrocytic expression of A_2B_R in the control OB (Figure 10R). However, the unchanged PCC value for A_2B_R-GFAP overlap and the increase in PCC value for A_2B_R/Iba1 signal overlap in EAE strongly suggested that the increase in A_2B_R expression in EAE occurred in infiltrated cells and/or reactive microglia (Figure 10R, GFAP/A_2B_R: *t* = 0.21, df = 39, *p* = 0.2172; Iba1/A_2B_R: *t* = 2.27, df = 37, *p* = 0.0377).

**Figure 11:**
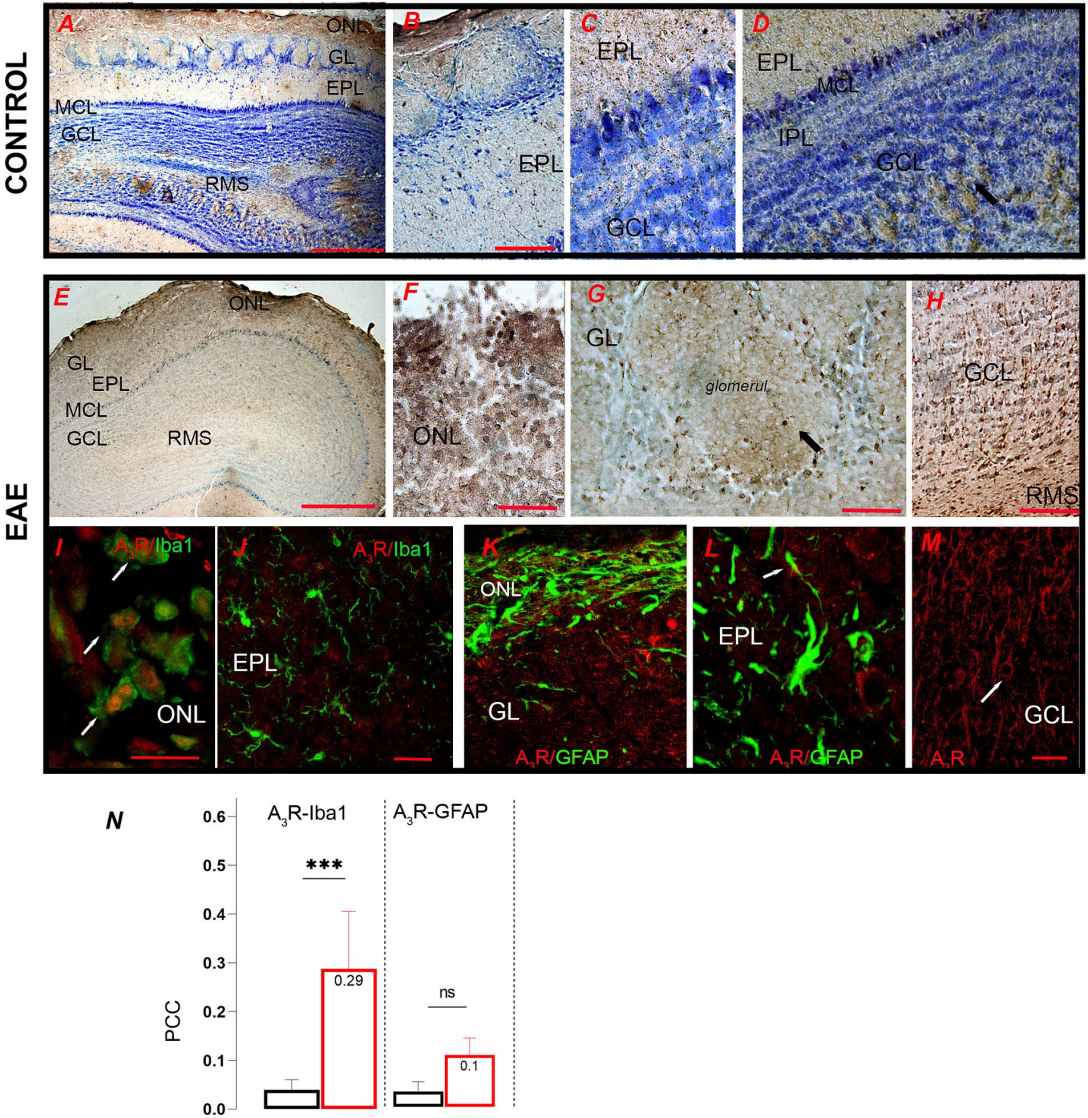
A_3_R-directed immunohistochemical labeling of the olfactory bulb. **(A)** Low-power microscopic image of control OB showing **(B)** strong A_3_R labeling in periglomerular neurons and **(C,D)** apical dendrites of mitral and tufted cells. **(E)** A_3_R-*ir* labeling in the EAE section shows **(F)** numerous A_3_R-*ir* infiltrates in the ONL and conspicuous labeling of **(G)** periglomerular neurons and **(H)** granular cells. **(I)** Double immunofluorescence directed against A_3_R (*red*) and Iba1 (*green*) shows dominant expression in infiltrated macrophages and ameboid microglia (*arrows*), and **(J)** the absence of A_3_R expression in ramified microglia in the EPL. **(K,L)** Double immunofluorescence directed to A_3_R (*red*) and GFAP (*green*) shows the absence of A3R expression in astrocytes. **(M)** Very pronounced A_3_R labeling of soma and apical dendrites of granule cells (arrow). **(N)** PCC value for A_3_R and Iba1 signal overlap in control (*black bars*) and EAE (*red bars*) sections. GCL, granule cell layer; GL, glomerular layer; EPL, external plexiform layer; IPL, internal plexiform layer; MCL, mitral cell layer; ONL, olfactory nerve layer; RMS—rostral migratory stream. Scale bar: 500 μm in **(A,E)**; 50 μm in **(B,C,D,F,G,H)**: 20 μm in **(I–M)**. Significance within the graph: ***p < 0.001.

With respect to A_3_R, the A_3_R-*ir* labeled periglomerular interneurons and granule cells (Figures 11A–C), whereas in the EPL, the apical dendrites of mitral and tufted cells extending perpendicular to the GL were most intensely stained (Figure 11D). The marked increase in A_3_R-*ir* in EAE (Figure 11E) was consistent with the mRNA and protein expression data. However, in contrast to control, numerous A_3_R-*ir* immune cells were seen in the ONL (Figure 11F). Periglomerular interneurons showed conspicuous A_3_R-*ir* (Figure 11G), while the most conspicuous A_3_R-*ir* was observed in GCL and RMS (Figure 11H). Double A_3_R/Iba1 immunofluorescence confirmed the dominant expression of A_3_R-*ir* in peripheral infiltrates and ameboid microglia in the ONL (Figure 11I), which was largely responsible for the observed A_3_R upregulation, while ramified microglia in the EPL lacked the A_3_R-*ir* (Figure 11J). Significant expression of A_3_R-*ir* was detected in granule cells in the GCL (Figure 11M). Double A_3_R/GFAP immunofluorescence showed low overlap between the two signals in ONL (Figure 11K) and EPL (Figure 11L). PCC value for A_3_R and Iba1 signal overlap was significantly increased in EAE compared to control (Figure 11N, *t* = 5.12, df = 37, *p* = 0.0006), confirming that infiltrated cells accounted for the increase in A_3_R-*ir* in EAE. On the other hand, the low PCC values for the signal overlap between A_3_R and GFAP indicate sparse localization and unaltered expression of A_3_R in astrocytes in EAE.

## 4 Discussion

Olfactory disorders often occur in neurodegenerative and psychiatric diseases and are retrospectively considered one of the earliest sensory symptoms (Bhatia-Dey and Heinbockel, 2021; Fatuzzo et al., 2023). A decrease in olfactory performance is common in patients with demyelinating autoimmune diseases such as MS and in the EAE animal model (Kim et al., 2018; Rotermund et al., 2019). The present study adds to the existing literature with the novel finding that reduced olfactory performance and impaired odor discrimination occur long before the clinical manifestation of EAE and that altered adenosine signaling may contribute to it. Regarding the first aspect, our study showed that the initial decline of odor detection and discrimination occurred at ~2 dpi, long before the appearance of the first motor signs, and the full pathological manifestation of EAE occurred at ~8–10 dpi. Interestingly, olfactory performance fluctuated with improvement and deterioration after the first episode at ~2 dpi, similar to early human RRMS. This finding suggests that the mechanism of olfactory impairment in EAE may recapitulate early pathologic events underlying human MS. Furthermore, our study has shown that animals in the early stages of EAE show behavioral changes indicative of anxiety, such as reduced general locomotion, altered exploratory behavior, and changes in odor-related social behavior, which are not due to motor impairments, but to olfactory dysfunction *per se*. This conclusion can be drawn based on the comparable OFT parameters in terms of locomotor behavior, distance traveled, and average and maximum speed of the EAE animals before and after immunization. The exact mechanisms of the behavioral changes likely involve neuroinflammation and changes in neurotransmitter systems (Tepavčević et al., 2011; Rotermund et al., 2019). These deficits may contribute to the deterioration of olfaction, as they may be related to the lack of motivation to explore and interact with novel objects due to sickness-like behavior, which is well documented in EAE (Pollak et al., 2003). When examining cognitive function, we also demonstrated that the reduced ability to discriminate social odors occurred concurrently with the deficit in novel object recognition, suggesting that this may be a consequence of impaired olfactory habituation and/or olfactory memory. Overall, the study clearly showed a significant impairment of olfaction as well as behavioral and cognitive changes in the early premotor phase of EAE.

**Figure 12:**
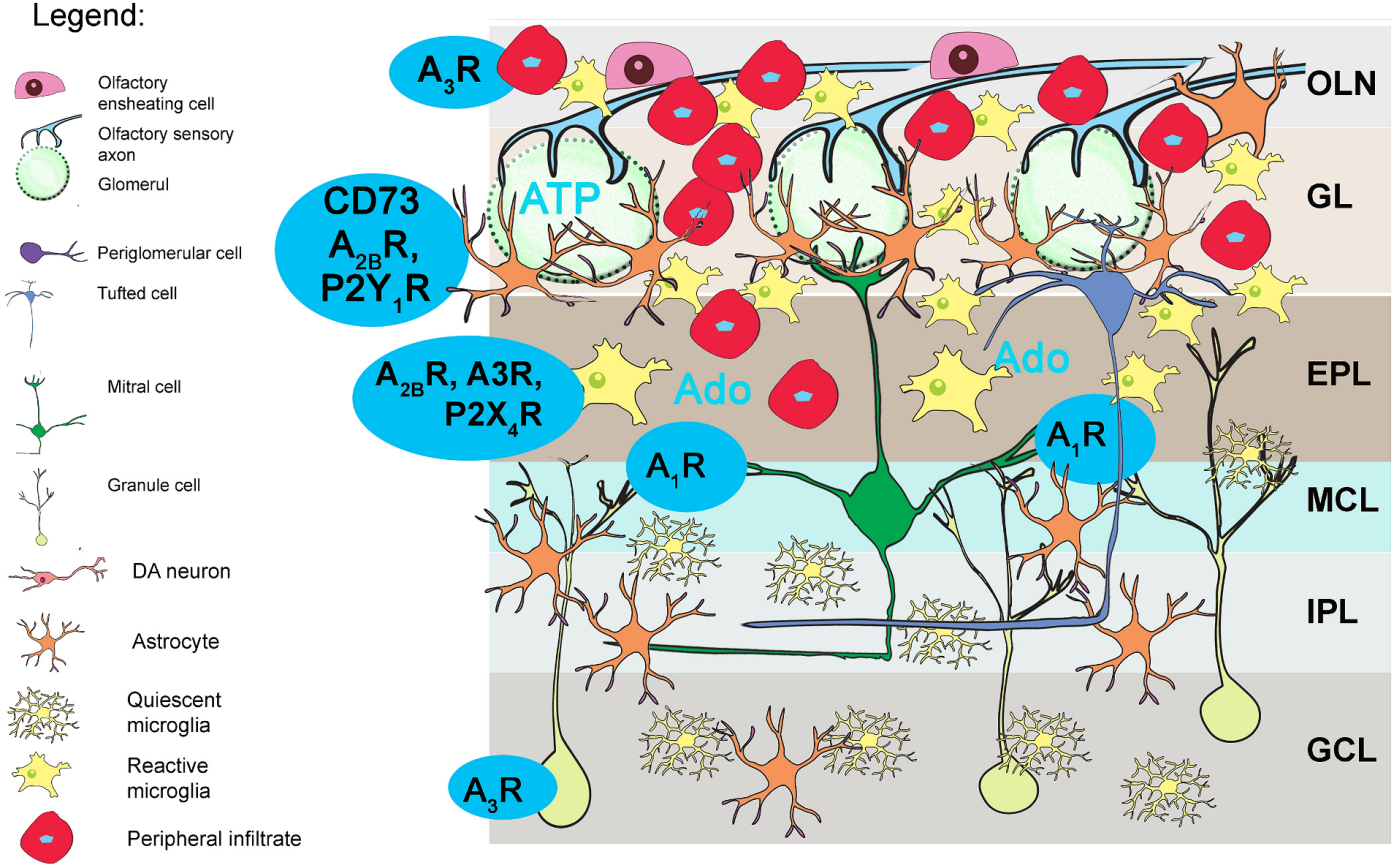
Purinergic signaling in OB in EAE. Schematic summary of the present study. The blue markers show the purinome components whose expression and cellular localization have changed significantly. Infiltrated macrophages and ameboid microglia overexpress A3R, while the latter also upregulates the expression of eN/CD73, A2BR, and P2X4R. Adenosine receptor A1R increased in mitral and tufted cells, likely influencing resting network activity in OB.

Although it is known that purinergic signaling plays a role in the pathophysiology of MS/EAE, there are still many unknowns about the contribution of the signaling system to olfactory dysfunction and behavioral changes in EAE. The olfactory bulb (OB) has an exceptionally high expression of purinergic receptors and ectonucleotidases, which are critically involved in olfactory information processing in the OB (Rotermund et al., 2019; Schubert et al., 2022; Wendlandt et al., 2023). The present study shows several significant changes in purinome expression in the OB during EAE (summarized in Figure 12), and their relationship with olfactory dysfunction can be considered by discussing two main aspects: (a) the information processing and plasticity of neuronal networks in the OB and (b) the neuroinflammatory response of infiltrated immune and resident glial cells.

### 4.1 Altered purinome expression in neural networks involved in information processing in EAE

The present study provided evidence for increased expression of A_1_R in EAE, mostly in mitral and tufted cells, where the receptor plays a functional role in neuronal circuits involved in the processing of odor information (Rotermund et al., 2019; Schubert et al., 2022; Wendlandt et al., 2023). Previous electrophysiological data show that tonic A_1_R activation in mitral cells is responsible for slow hyperpolarizing oscillations of mitral cell membrane potential (Roux et al., 2015), which control glutamate release at their dendro-dendritic synapses with granule cells (Rotermund et al., 2018; Schulz et al., 2018). As a result, the granule cells reduce GABA release in the reciprocal synapses and attenuate recurrent mitral cell inhibition (Rotermund et al., 2018). Therefore, tonic A_1_R activity provides a high output signal-to-noise ratio during olfactory stimulation (Rotermund et al., 2018). Therefore, it can be assumed that any deviation from baseline A_1_R activity impacts the entire network via modulation of reciprocal synapses between mitral and granule cells. This assumption is supported by data showing that stimulation of granule cells enhances odor discrimination, while their inhibition impairs it (Abraham et al., 2010; Gschwend et al., 2015). Accordingly, A_1_R-deficient mice have shown better olfactory performance compared to their wild-type counterparts (Schulz et al., 2018). On the other hand, A_1_Rs have no effect on mitral cell bursting activity upon synaptic input from olfactory sensory axons (Rotermund et al., 2018, 2019). Instead, glomerular synaptic arrangements between olfactory sensory axons and mitral cell dendrites are regulated by ATP and adenosine via P2Y_1_R and A_2A_R, respectively, which are mainly expressed in periglomerular astrocytes and interneurons (Johansson et al., 1997; Kaelin-Lang et al., 1998). Our data showed no changes in gene expression for *Adora2a* or *P2y1r*, confirming the recent finding that they are not involved in EAE-induced impairment of olfaction (Wendlandt et al., 2023).

Our study also found the reduction in total TH-immunohistochemical labeling in the GL. About 10–16% of all GL interneurons express TH, and among them, the most numerous are axonless periglomerular interneurons with small cell body and dendrites extending to the neighboring glomerulus, and short axon surface (sSA) cells, which have a long axon extending to a variable number of glomeruli. Determination of the integrated TH-ir density showed that the decrease in TH-*ir* is due to the apparently weaker reactivity of neurites extending within and between glomeruli. DA neurons play an important role in odor processing as they inhibit excitatory transmission to OB excitatory (or principal) neurons (Borisovska et al., 2013; Banerjee et al., 2015; Liu et al., 2016; Capsoni et al., 2021). However, counting the number of TH^+^ cell bodies unexpectedly revealed an increased density of TH^+^ cell bodies in the GL. Therefore, it can be assumed that the reduced TH^+^ reactivity of DA neurites and the higher number of TH^+^ cells probably influence the resting activity of mitral cells in EAE animals. In healthy animals, periglomerular cells are continuously replaced by type A cells arriving from the SVZ via the RMS to OB. However, a previous study performed in EAE indicated that impaired migration of neuroblasts in the RMS may lead to reduced replacement of periglomerular cells in the OB (Tepavčević et al., 2011). Indeed, in our study, a reduced number of type A and type B neuroblasts was found in the RMS, along with a marked atrophy of granule cells in the GCL. Therefore, our data do not suggest an increased incorporation of new periglomerular cells in the GL, but rather some other compensatory mechanism(s), including cell type-specific enhancement of TH^+^ cell neurogenesis or altered fate specification. DA neurons are involved in substantial activity-dependent plasticity, which impacts the cell composition of DA population in OB through modulation of adult neurogenesis (Bovetti et al., 2009). Furthermore, the level of TH expression and dopamine release depend on the sensory input (for review, see Bonzano et al., 2016). While the molecular mechanisms of the activity-dependent plasticity of DA neurons remain to be elucidated, it is possible that peripheral infiltrates and reactive microglia release inflammatory mediators that affect newcomers arriving in the GL during EAE. Among other inflammatory mediators, it was shown that IL-6, which increased significantly in EAE in OB, plays a crucial role in adult DA neurogenesis and differentiation in OB (Storer et al., 2018). Taken together, the data suggest that the enhancement of A_1_R-mediated signaling in principal output OB neurons and the altered connectivity of DA neurons likely influence resting network activity and contribute to impaired odor processing in EAE.

### 4.2 Purinergic signaling in neuroinflammatory response of infiltrated immune cells and resident glia

Regarding neuroinflammation, our study showed massive infiltration of the OB with immune cells and marked microgliosis in EAE, which are otherwise recognized as the cause of the early inflammatory lesions in MS/EAE (Kaskow and Baecher-Allan, 2018). A previous comprehensive assessment of the spatiotemporal pattern of CNS infiltration has shown that the initial recruitment of CD4^+^ T cells in EAE occurs via the meninges and choroid plexus rather than the vasculature (Brown and Sawchenko, 2007). While our data confirmed the presence of CD4^+^ T cells and monocytes/macrophages in the superficial layers of the OB in contact with the subarachnoid space, the presence of infiltrates in the subependymal zone and RMS could not be detected. The spatial arrangement of reactive microglia mirrored that of CD4^+^ T cells, with a decreasing gradient from the superficial to the innermost layers of the OB. Our additional observation that infiltration occurred more frequently on the lateral side than on the medial side of the OB supports the conclusion that the subarachnoid space was the main route for immune cell entry in the OB, at least at this stage of EAE. A recent study reported a structural feature that may predispose the OB as one of the earliest targets in MS/EAE (Fatuzzo et al., 2023; Spera et al., 2023). In particular, anatomical discontinuity of the arachnoid barrier at the level of the cribriform plate on the CNS side could be the cause of early infiltration of the OB with immune cells (Spera et al., 2023).

Our data show that infiltrated monocytes/macrophages abundantly express A_3_R. Induction of *Adora3* and A_3_R has been previously demonstrated in peripheral lymphoid (Wei et al., 2013) and inflamed spinal cord tissue during EAE (Wei et al., 2013; Jakovljevic et al., 2017), as well as during ischemia, hypoxia, and subarachnoid hemorrhage (Coppi et al., 2023). Since A_3_R controls neutrophils and T-cell activity (Butler et al., 2012; Gessi et al., 2013) and M1/M2 polarization in macrophages (Levy et al., 2006; Li et al., 2020), it may be also responsible for functional phenotype of activated microglia. Specifically, A_3_R-mediated signaling controls the release of inflammatory cytokines, including TNF-α via p68/STAT6 pathway, thus contributing to development of functional phenotype of macrophages and microglia (Coppi et al., 2022).

The pattern of peripheral immune cell infiltration overlapped with the pattern of microgliosis. A significantly higher number of microglial cells was found in GL, EPL, and GCL, while their reactivity decreased with increasing distance from the lateral side of the OB. Microgliosis was induced by the cytokines of CD4^+^ T cells, i.e., IL-17 and IFN-γ, which promote inflammation by inducing local expression of proinflammatory cytokines by reactive microglia (Dogan and Karpus, 2004; Sindern, 2004; Murphy et al., 2010). Indeed, the present study reported several-fold induction of TNF-α, IL-1β, and IL-6, which are likely responsible for the observed changes in purinergic signaling (Adzic Bukvic et al., 2024). Several previous studies showed a significant increase in eN/CD73 (Lavrnja et al., 2015; Jakovljevic et al., 2017), A_2A_R (Jakovljevic et al., 2017), A_2B_R (Wei et al., 2013), and A_3_R (Wei et al., 2013; Jakovljevic et al., 2017) in inflamed spinal cord tissue in EAE. The present study shows no changes in A_2A_R expression in the OB, which is consistent with stable A_2A_R signaling in the OB in EAE (Wendlandt et al., 2023). However, we have shown that highly reactive microglial cells express eN/CD73, A_2B_R, and A_3_R, which are not present in quiescent microglial cells under physiological conditions (Langer et al., 2008; Franco et al., 2021). Under basal conditions, eN/CD73 is localized in astrocytes and ependymal cells, while under neuroinflammatory conditions, it is expressed in ameboid microglia overexpressing arginase 1 and CD68 (Dragić et al., 2021b), characterizing the phagocytic microglial state (Franco and Fernández-Suárez, 2015). As for A_2B_R, it is localized in astrocytes, oligodendrocytes, and neurons under physiological conditions, while it is expressed in reactive microglia during neuroinflammation. A_2B_R stimulates the production of IL-6 in peripheral immune cells and plays pathogenic roles in MS/EAE by enhancing Th17 differentiation (Wei et al., 2013; Merighi et al., 2017) and functional polarization of microglial cells (Koscso et al., 2012; Franco et al., 2021). As for A_3_R, activation in reactive microglia alters the expression of numerous immune-related genes, including the attenuation of TNF-α (Lee et al., 2006) and the increase in IL-10 production (Haskó et al., 1996, 2008). Our data also showed transcriptional upregulation of *P2xr4* in EAE, which is the key determinant in the control of microglial activation and induction of microglial cell death (Vázquez-Villoldo et al., 2014). The A_2B_R and A_3_R are coupled to Gs and Gi/q, respectively, and therefore influence adenylate cyclase and cAMP levels in opposite ways (Fredholm et al., 2011). However, both receptors mobilize Ca^2+^ from intracellular stores via phospholipase C (Fredholm et al., 2011). Stimulation of P2X_4_R in microglia also leads to an increase in intracellular Ca^2+^ and activation of p38-MAPK (Trang et al., 2009). Therefore, the simultaneous activation of A_2B_R, A_3_R, and P2X_4_R in cells such as reactive microglia may not be contradictory from a physiological point of view. Overall, these data, together with the finding of increased expression of C3, involved in phagocytizing complement-opsonized synapses (Hammond et al., 2020), suggest that OB microglial cells in EAE develop a functional phenotype of phagocytizing microglia that promotes tissue repair and homeostasis. In conclusion, the exceptionally high abundance of purinergic receptors and nucleotide-degrading enzymes in OB underscores the importance of purinergic signaling for the olfactory system, and the present study adds to the existing knowledge on the contribution of adenosine signaling to the impairment of olfaction in EAE. Our results suggest enhanced adenosine signaling via A_1_R in principal OB neurons, which may alter resting network activity in EAE. Our data further show enhanced adenosine signaling in microglial cells via the eN/CD73/A_2B_R/A_3_R molecular pathway(s) and induction of inflammatory mediators that may be responsible for the impairment of olfaction in EAE and likely in other CNS disorders. It is worth noting, however, that the present study did not address the causal relationship between these phenomena. However, not only does adenosine signaling contribute to the causative processes that lead to the main neurological and motor symptoms in MS/EAE, but the signaling has been linked to development of neuropsychiatric comorbidities characteristic of MS, such as anxiety and depression (Duarte-Silva et al., 2019; Gomes et al., 2021). Therefore, some degree of overlap and dysfunction in the adenosine signaling pathway would be expected as the mechanism responsible for the apparent neuroinflammation, olfactory dysfunction, and anxiety-related behavioral changes. However, further functional studies in genetic models are required for a complete assessment. Further investigation of olfactory dysfunction in MS/EAE may provide a valuable window for monitoring the disease onset and progression, while investigation of the functional role of purinergic signaling may point to potential molecular targets for therapeutic intervention in MS.

## Data availability statement

The original contributions presented in the study are included in the article/Supplementary material, further inquiries can be directed to the corresponding author.

## Ethics statement

The animal study was approved by the Ethics Committee for the Care and Use of Laboratory Animals of the Faculty of Biology of the University of Belgrade. The study was conducted in accordance with the local legislation and institutional requirements.

## Author contributions

AS: Formal analysis, Investigation, Visualization, Writing – original draft, Writing – review & editing. MD: Funding acquisition, Validation, Visualization, Writing – review & editing, Formal analysis, Methodology, Project administration, Resources. JS: Resources, Writing – review & editing, Investigation. MZK: Investigation, Writing – review & editing. IS: Writing – review & editing, Resources. MZJ: Writing – review & editing, Investigation. KM: Investigation, Writing – review & editing. NN: Writing – review & editing, Conceptualization, Data curation, Funding acquisition, Supervision, Validation, Visualization.

## Footnotes

^1^https://imagej.net/ij/plugins/cell-counter.html
