# Supplementary material for: Impaired olfactory performance and anxiety-like behavior in a rat model of multiple sclerosis are associated with enhanced adenosine signaling in the olfactory bulb via A1R, A2BR, and A3R
Source: Front Cell Neurosci. 2024 Jul 30;18:1407975. doi: 10.3389/fncel.2024.1407975 (PMC11320153; doi:10.3389/fncel.2024.1407975)
Supplement: Supplementary file 1 [file Table_1.DOCX]

Supplementary Material

IMPAIRED OLFACTORY PERFORMANCE AND ANXIETY-LIKE BEHAVIOR IN A RAT MODEL OF MULTIPLE SCLEROSIS ARE ASSOCIATED WITH ENHANCED ADENOSINE SIGNALING IN THE OLFACTORY BULB VIA A1R, A2BR AND A3R First Author*, Co-

Andjela Stekic^1^, Milorad Dragic^1^, Jelena Stanojevic^2^, Marina Zaric ^3^, Ivana Stevanovic^2^, Milica Zeljkovic Jovanovic^1^, Katarina Mihajlovic^1^, Nadezda Nedeljkovic^1,*^

*** Correspondence:** Nadezda Nedeljkovic: nnedel@bio.bg.ac.rs

# Supplementary Figures and Tables

## Supplementary Figure *S1*


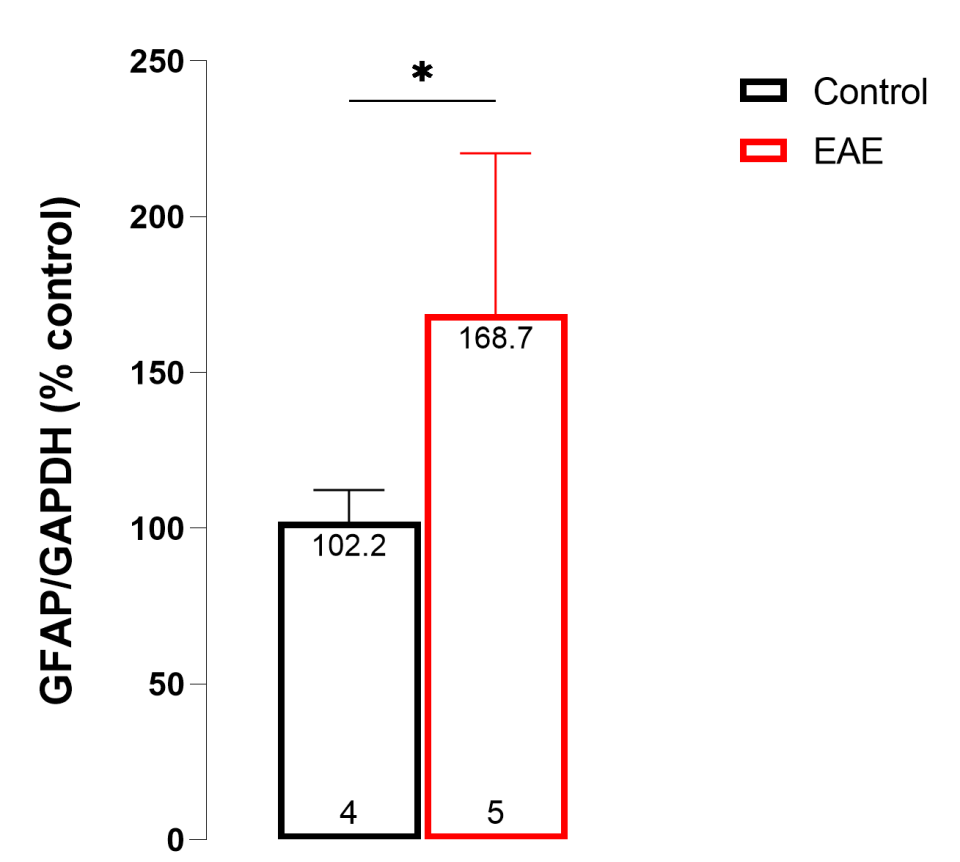


## Legend to Supplementary Figure *S1*. Relative GFAP/GAPDH protein abundance as determined by Western blotting in P2 membrane fractions isolated from OB tissue obtained from control (*black bar*) and EAE animals (*red bar*). Numbers inside the bars denote the number of animals in each group.

##
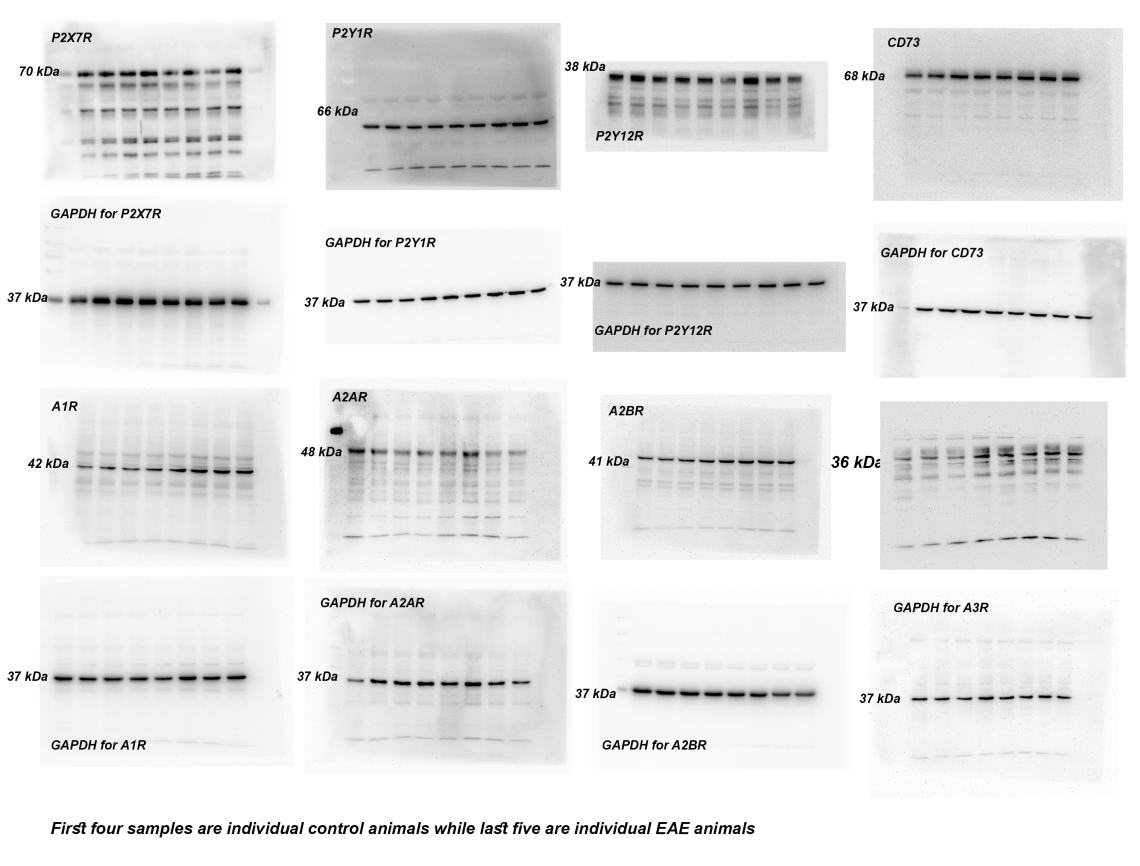
Supplementary Figure *S2*

**Legend to Supplementary Figure *S2*.** Non-cropped PVDF support membranes presented in Figure 7. Lanes 1-4 - P2 samples isolated from control OB, lanes 5-8 – four P2 samples isolated from EAE animals.

## Supplementary Figure *S*3


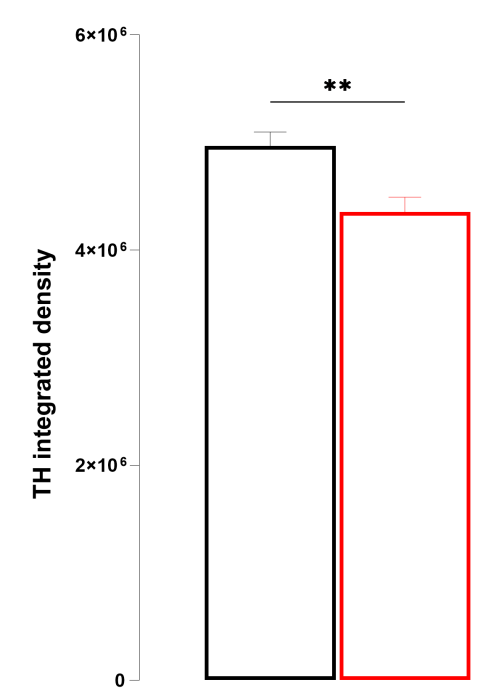


A.


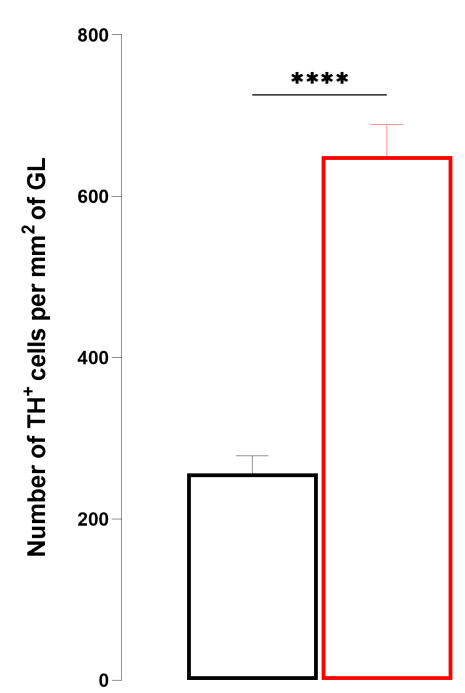


B.

Legend to Supplementary Figure *S3*. Graphs showing the TH-*ir* integrated density (A) and the number of TH-immunoreactive cells in the GL layer in control (*black bars*) and EAE (*red bars*). Quanatification was performed in ImageJ, as described in Materials and methods Section.

**1.4. Supplementary Figure *S4***


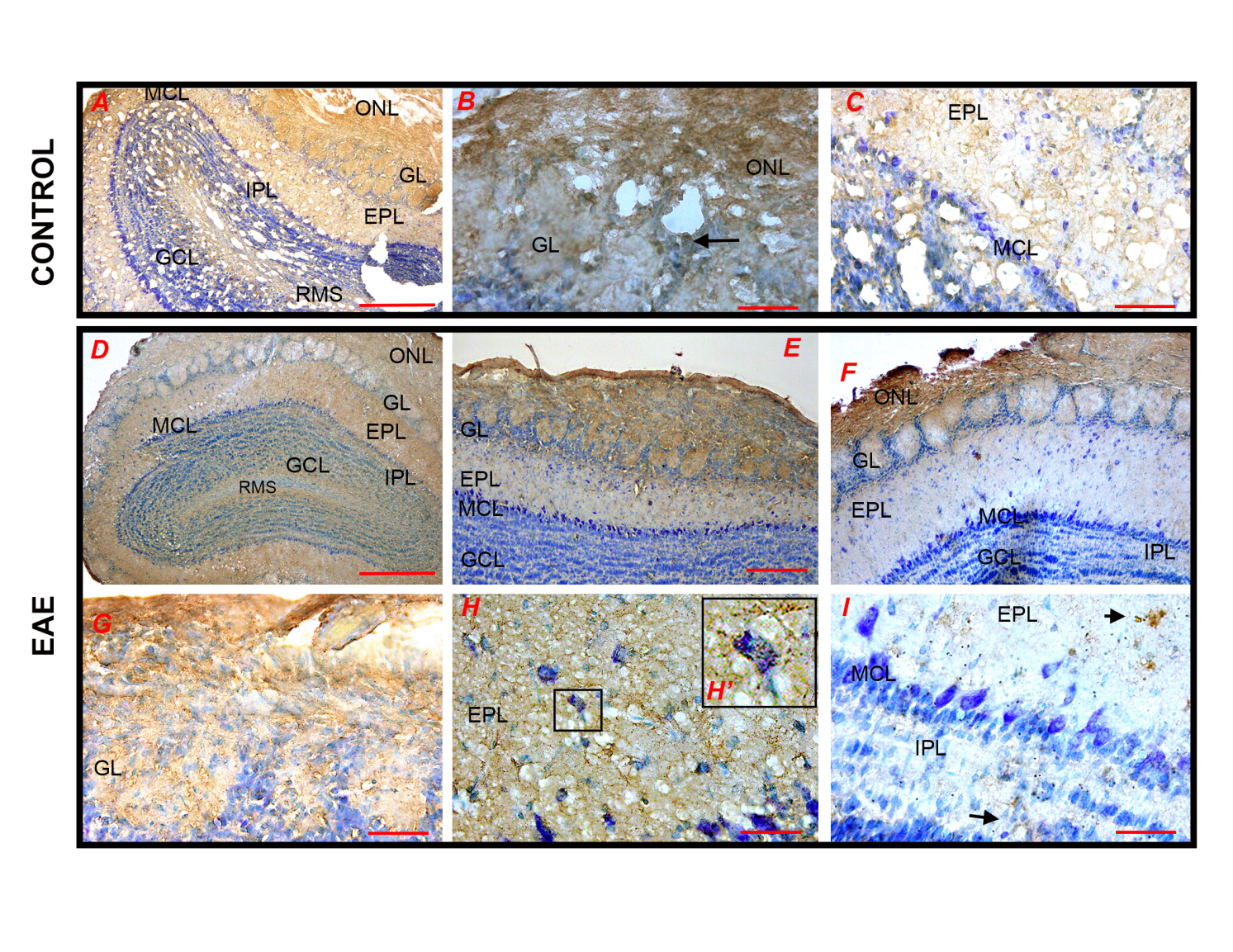
**Legend to Supplementary Figure *S*4.** A_2A_R-directed immunohistochemical labeling of the olfactory bulb. A) Low-power photomicrograph of control OB, showing A2AR-ir in ONL, B) periglomerular neurons (arrow), C) EPL parenchyma and MCL. D) The overall pattern of A2AR-ir is comparable in EAE and control. E) A2AR-ir in GL, F) EPL and I) GCL (arrow) corresponds to astrocytic expression. (I, arrow). H) Conspicuous neuronal labeling in the tufted cells (enlarged in H’). Abbreviations: GCL, granule cell layer; GL, glomerular layer, EPL, external plexiform layer; IPL, internal plexiform layer; MCL, mitral cell layer; ONL, olfactory nerve layer, RMS – rostral migratory stream. Scale bar: 500 µm in A, D; 100 µm in E, F; 50 µm in B, C, G, H, I.

# Supplementary Tables

## Supplementary Table S1

| **Table *S1***. Epidemiological data^*^ | | |  |
| --- | --- | --- | --- |
| **Parameter** | | **Control** | **EAE** |
| Total number of animals | | 90 | |
| No. of animals in experimental group | | 18 | 66 |
| Number of experimental units | | 5 | 17 |
| Animals exluded from the study | | 0 | 6 |
| Mean body mass at the begining of study (g) | | 184.6 ± 20.4 | 192.8 ± 14.4 |
| Mean body mass at sacrifice (g) | | 197.3 ± 19.2 | 183.1 ± 0.1 |
| Incidence of EAE | | / | 65/66 (98.4%) |
| Mean dpi to onset | | / | 8.1 ± 1.2 |
| Mean dpi to sacrifice | |  | 11.9 ± 1.4 |
| Maximum mean score (at 12-13 dpi) | | / | 2.0±1.1 |
| Mortality | | / | 0/28 |
| Baseline latency in BFT (s) | 182.4 ±42.4 | 192.2±54.0 |  |
| *Parameters of EAE and baseline latency in BFT were determined as described in Materials and methods. Data are presented as mean ± SEM. | | |  |

| Supplementary Table *S*2 **Table *S2*. List of primary and secondary antibodies used in the study** | | | | |
| --- | --- | --- | --- | --- |
| Epitope | Source and clonality | Dilution and application | Manufacturer | RRID |
| GFAP | Rabbit, *pc*  Chicken, *pc* | 1:500^IHC^  1:400^IF^ | DAKO  Novus Biologicals | RRID:AB_10013382 RRID: AB_1556315 |
| Iba1 | Goat, *pc* | 1:600^IHC^  1:400^IF^ | Abcam, ab5076, | RRID:AB_2224402 |
| CD4 | Mouse, *pc* | 1:100^IHC^ | Abcam | RRID:AB_2828023 |
| DCX | Goat, *pc* | 1:200^IHC^ | Santa Cruz Biotechnology | RRID:AB_2088494 |
| TH | Rabbit, *pc* | 1:500^IHC^ | Millipore | RRID:AB_390204 |
| NeuN | Mouse, *pc* | 1:200^IHC^ | Millipore | RRID:AB_2313673 |
| P2X_7_R | Rabbit, *pc* | 1:1000^WB^ | Alomone | RRID:AB_2040068 |
| P2Y_1_R | Rabbit, *pc* | 1:1000^WB^ | Alomone | RRID:AB_10919250 |
| P2Y_12_R | Rabbit, *pc* | 1:500^WB^ | Alomone | RRID:AB_2040074 |
| NTPDase2 | Rabbit, *pc* | 1:300^IHC^ | ectonucleotidases-ab.com | RRID:AB_2314986 |
| eN/CD73 | Rabbit, *pc*  Rabbit, *pc* | 1:1500^WB^  1:300^IHC^ | Cell Signalling Technology  Ectonucleotidases-ab-com | RRID:AB_2716625  rNu-9 L(I4,I5) |
| ADA | Rabbit, *pc* | 1:2000^WB^ | ThermoFisher Scientific | RRID:AB_2637694 |
| A_1_R | Rabbit, *pc* | 1:1000^WB^, 1:200^IHC^  1:200^IF^ | Alomone | RRID:AB_2039705 |
| A_2A_R | Rabbit, *pc* | 1:1000^WB^  1: 200^IHC^ | ThermoFisher Scientific | RRID:AB_2257858 |
| A_2B_R | Rabbit, *pc* | 1:1000^WB^, 1:200^IHC^  1:200^IF^ | Alomone | RRID:AB_2039709 |
| A_3_R | Rabbit, *pc* | 1:1000^WB^, 1:100^IHC^  1:200^IF^ | Alomone | RRID:AB_2039711 |
| GAPDH | Rabbit, *pc* | 1:2000^WB^ | Invitrogen | RRID:AB_2107311 |
| β - Actin | Mouse, *pc* | 1:5000^WB^ | Abcam | RRID: AB_867494 |
| Goat IgG (H+L) | Donkey, pc Alexa Fluor 488 | 1:400^IF^ | Invitrogen | RRID: AB_142672 |
| Rabbit IgG  (H+L) | Donkey, pc  Alexa Fluor 555 | 1:400^IF^ | Invitrogen | RRID: AB_141784 |
| Mouse IgG  (H+L) | Donkey, pc  Alexa Fluor 488 | 1:400^IF^ | Invitrogen | RRID:AB_141607 |
| Chicken IgG (H+L) | Goat, pc  Alexa Fluor 488 | 1:400^IF^ | Invitrogen | RRID:AB_142924 |
| Rabbit IgG  (H+L) | Goat, pc  HRP conjugated | 1:30 000^WB^, 1:2000^IHC^ | Abcam | RRID: AB_955447 |
| Goat IgG  (pAB) | Rabbit, pc  HRP conjugated | 1:200^IHC^ | R&D | RRID:AB_562588 |

## Supplementary Table *S*3

| Table *S3*. List of primer pairs used for RT-qPCR | | |  |
| --- | --- | --- | --- |
| Target gene | | *Forward* | *Reverse* |
| *Ada* | GAGCCTCATCCTGTGAATGG | ATGCCCATGATTGTCAAGGT |  |
| *Adora1* | GTGATTTGGGCTGTGAAGGT | TCTGTGGCCCAATGTTGATAAG |  |
| *Adora2a* | TGCAGAACGTCACCAACTTC | CAAAACAGGCGAAGAAGAGG |  |
| *Adora2b* | CGTCCCGCTCAGGTATAAAG | CCAGGAAAGGAGTCAGTCCA |  |
| *Adora3* | TTCTTGTTTGCCTTGTGCTG | AGGGTTCATCATGGAGTTCG |  |
| *Cy3* | GCGGTACTACCAGACCATCG | CTTCTGGCACGACCTTCAGT |  |
| *Ent1* | CACTTCCTTCGCTGTTAGGG | TGTCCCCCTACCACTCTGAC |  |
| *Ent2* | CCCTCATGACCTTCTTCCTG | CCAAGAGACCCGGTATAGCA |  |
| *Entpd1* | TCAAGGACCCGTGCTTTTAC | TCTGGTGGCACTGTTCGTAG |  |
| *Entpd2* | TGCTTCGACACAGATCACCT | GAATCTGGTCTCGGCCATAG |  |
| *Gapdh* | TGGACCTCATGGCCTACAT | GGATGGAATTGTGAGGGAGA |  |
| *Il1b* | AAACAGCAATGGTCGGGACA | GTCCTGGGGAAGGCATTAGG |  |
| *Il6* | CCGGAGAGGAGACTTCACAG | ACAGTGCATCATCGCTGTTC |  |
| *Lcn2* | GGATCAGAACATTCGTTCCA | GGATGGAATTGTGAGGGAGA |  |
| *Nt5e* | CAAATCTGCCTCTGGAAAGC | ACCTTCCAGAAGGACCCTGT |  |
| *P2rx2* | TGGGACTACGAGACGCCTAA | CTCGCTGTCCTGGTAGCTTT |  |
| *P2rx4* | CGTGGCGGACTATGTGATTC | GGAATCTCTGGACAGGTGCT |  |
| *P2rx7* | ATTGTTAGGCCAATGGCAAG | AACACCTTCACCGTCTCCAC |  |
| *P2ry1* | CTGGATCTTCGGGGATGTTA | CTGCCCAGAGACTTGAGAGG |  |
| *P2ry12* | CGAAACCAAGTCACTGAGAGGA | CCAGGAATGGAGGTGGTGTTG |  |
| *Tnfa* | CTCCCAGAAAAGCAAGCAAC | CGAGCAGGAATGAGAAGAGG |  |

## Supplementary Table *S*4

| **Supplementary Table *S*4. Parameter obtained from open-field test** | | |
| --- | --- | --- |
| **Parameter** | **Control** | **EAE** |
|  |  | Student’s *t* test/Wilcoxon matched-pairs signed rank test |
| Number of entries in central fields | 1.4 ± 1.4 | 0.7 ± 1.1 |
|  | *(t* = 0.9575, d_f_ = 9, *p* = 0.363) | |
| Time spent in central fields (s) | 13.0 ± 22.4 | 3.0 ± 5.0 |
|  | (W = -23, *p* = 0.275) | |
| Distance travelled in central fields (m) | 0.7 ± 0.7 | 0.3 ± 0.6 |
|  | (W = -21, *p* = 0.322) | |
| Latency to central fields entry (sec) | 179 ± 191 | 95 ± 163 |
|  | (W = -15, *p* = 0.492) | |
| Average speed in central fields (m/s) | 0.05 ± 0.06 | 0.03 ± 0.05 |
|  | *(t* = 0.6359, d_f_ = 9, *p* = 0.541) | |
| Maximal speed in central fields (m/s) | 0.20 ± 0.18 | 0.13 ± 0.17 |
|  | *(t* = 0.6482, d_f_ = 9, *p* = 0.533) | |
| Mobile time in central fields (s) | 13.0 ± 22.3 s | 3.0 ± 5.0 s |
|  | *(t* = 1.277, d_f_ = 9, *p* = 0.233) | |
| Number of entries in peripheral fields | 51.3 ± 18.5 | 2.3 ± 1.7 |
|  | *(t* = 8.259, d_f_ = 9, *p* < 0.0001*) | |
| Time spent in peripheral fields (s) | 457 ± 44 | 583 ± 34 |
|  | (W = 55, *p* = 0.002*) | |
| Distance travelled in peripheral fields (m) | 16.6 ± 4.6 | 12.6 ± 5.1 |
|  | *(t* = 2.615, d_f_ = 9, *p* = 0.028*) | |
| Average speed in peripheral fields (m/s) | 0.04 ± 0.01 | 0.02 ± 0.01 |
|  | *(t* = 4.118, d_f_ = 9, *p* = 0.0026*) | |
| Maximal speed in peripheral fields (m/s) | 0.52 ± 0.11 | 0.48 ± 0.11 |
|  | *(t* = 1.113, d_f_ = 9, *p* = 0.2944) | |
| Mobile time spent in peripheral fields (s) | 450 ± 51 | 475 ± 74 |
|  | *(t* = 1.149, d_f_ = 9, *p* = 0.2803) | |
| Immobile time spent in peripheral fields (s) | 6.7 ± 14.2 | 108.7 ± 74.1 |
|  | (W = 45, *p* = 0.0039*) | |
| Number of immobile episodes in peripheral fields | 0.2 ± 0.4 | 1.9 ± 1.1 |
|  | (W = 45, *p* = 0.0039*) | |
| Number of freezing episodes in peripheral fields | 4.7 ± 8.3 | 8.3 ± 2.7 |
|  | (W = 0, p > 0.9999) | |
| Freezing time spent in peripheral fields (s) | 17.5 ± 17.6 | 44.8 ± 40.5 |
|  | (*t* = 1.975, d_f_ = 9, *p* = 0.079) | |
| Number of entries in corners | 26.6 ± 7.8 | 13.4 ± 5.5 |
|  | *(t* = 5.88, d_f_ = 9, *p* = 0.0002*) | |
| Time spent in corners (s) | 360 ± 54 | 524 ± 44 |
|  | *(t* = 7.808, d_f_ = 9, *p* < 0.0001*) | |
| Average speed in corners (m/s) | 0.02 ± 0.01 | 0.01 ± 0.00 |
|  | (*t* = 6.447, d_f_ = 9, *p* = 0.0001) | |
| Maximal speed in corners (m/s) | 0.44 ± 0.10 | 0.46 ± 0.13 |
|  | (*t* = 0.2916, d_f_ = 9, *p* = 0.777) | |
| Mobile time spent in corners (s) | 353 ± 60 s | 415 ± 64 |
|  | (*t* = 2.676, d_f_ = 9, *p* = 0.0254*) | |
| Immobile time spent in corners (s) | 6.7 ± 14.2 | 108.7 ± 74.0 |
|  | (W = 45, *p* = 0.0039*) | |
| Number of immobile episodes in corners | 0.2 ± 0.4 | 1.9 ± 1.9 |
|  | (W = 45, *p* = 0.0039*) | |
| Number of freezing episodes in corners | 1.2 ± 1.2 | 3.0 ± 2.7 |
|  | W = 22, *p* = 0.0625 | |
| Freezing time spent in corners (s) | 17.0 ± 17.8 | 44.8 ± 40.5 |
|  | (*t* =2.010, d_f_ = 9, *p* = 0.0753) | |

## Supplementary Table *S*5.

| **Table *S*5. Novel object recogntion test data** | | | | | |
| --- | --- | --- | --- | --- | --- |
| **Parameter** | **Control** | | | **EAE** | |
|  | Student’s *t* test/  Wilcoxon matched-pairs signed rank test | | | | |
| FO1 number investigations | 13.6 ± 9.2 | | | 2.2 ± 3.1 | |
|  | *(t* = 4.419, d_f_ = 11, *p* = 0.001*) | | | | |
| Investigation time in the FO1 zone (s) | 20.7 ± 13.1 | | | 3.7 ± 5.6 | |
|  | W = -78, *p* = 0.0005* | | | | |
| Distance travelled in the zone of FO1 (m) | 0.44 ± 0.31 | | | 0.11 ± 0.15 | |
|  | W = -66, *p* = 0.001* | | | | |
| Distance travelled before first investigating in the zone of FO1 (m) | 3.7 ± 3.8 | | | 1.5 ± 1.9 | |
|  | *(t* = 1.677, d_f_ = 11, *p* = 0.1218*) | | | | |
| Latency to first investigation in the zone of FO1 (s) | 120.2 ± 137.4 | | | 75.0 ± 143.3 | |
|  | W = -24, *p* = 0.3804 | | | | |
| Average speed while investigating in the zone of FO1 (m/s) | 0.02 ± 0.01 | | 0.03 ± 0.05 | | |
|  | W = -5, *p* = 0.8486 | | | | |
| Mobile time spent investigating the zone of FO1 (s) | 18.8 ± 9.3 | | 3.7 ± 5.6 | | |
|  | W = -78, *p* = 0.0005* | | | | |
| Head average distance from the zone of FO1 (m) | 0.42 ± 0.12 | | 0.49 ± 0.25 | | |
|  | (*t* = 0.7711, d_f_ = 11, *p* = 0.4569) | | | | |
| Head max distance from the zone of FO1 (m) | 0.95 ± 0.05 | | | 0.81 ± 0.20 | |
|  | W = -60, *p* = 0.0161* | | | | |
| Head min distance from the zone of FO1 (m) | 0 ± 0 m | | | 0.1 ± 0.1 | |
|  | W = 21, *p* = 0.0313* | | | | |
| Time spent oriented to the zone of FO1 (s) | 138.8 ± 43.0 | | 176.9 ± 96.5 | | |
|  | *(t* = 1.43, d_f_ = 11, *p* = 0.1805) | | | | |
| FO2 number investigations | 5.2 ± 4.2 | | | 3.5 ± 4.1 | |
|  | W = -30, *p* = 0.2544 | | | | |
| Investigation time in the FO2 zone (s) | 8.2 ± 6.5 | | | | 4.6 ± 4.8 |
|  | *t* = 1.333, d_f_ = 11, *p* = 0.2096 | | | | |
| Distance travelled in the zone of FO2 (m) | 0.25 ± 0.20 | | 0.15 ± 0.13 | | |
|  | *(t* = 1.5, d_f_ = 11, *p* = 0.1618) | | | | |
| Distance travelled before first investigating in the zone of FO2 (m) | 5.6 ± 4.4 | | | 3.1 ± 2.7 | |
|  | *(t* = 0.8124, d_f_ = 4, *p* = 0.4622) | | | | |
| Latency to first investigation in the zone of FO2 (s) | 114.8 ± 103.4 | | | 182.9 ± 198.3 | |
|  | *(t* = 1.72, d_f_ = 4, *p* = 0.1605) | | | | |
| Average speed while investigating in the zone of FO2 (s) | 0.03 ± 0.01 | | 0.04 ± 0.02 | | |
|  | *(t* = 1.466, d_f_ = 4, *p* = 0.2166) | | | | |
| Mobile time spent investigating the zone of FO2 (s) | 8.2 ± 6.5 | | 4.6 ± 4.8 | | |
|  | *(t* = 0.8583, d_f_ = 11, *p* = 0.4091) | | | | |
| Head average distance from the zone of FO2 | 0.62 ± 0.10 | 0.58 ± 0.22 | | | |
|  | *(t* = 0.6062, d_f_ = 11, *p* = 0.5567) | | | | |
| Head max distance from the zone of FO2 (m) | 0.93 ± 0.01 | 0.82 ± 0.12 | | | |
|  | W = -78, *p* = 0.0005* | | | | |
| Head min distance from the zone of FO2 (m) | 0.04 ± 0.07 | | 0.05 ± 0.10 | | |
|  | W = 2, *p* = 0.9219 | | | | |
| Time spent oriented to the zone of FO2 | 139.8 ± 47.63 | | | 188.7 ± 102.4 | |
|  | *(t* = 1.726, d_f_ = 11, *p* = 0.1123) | | | | |
| FO1 (2) number investigations | 10.4 ± 6.5 | | | 8.8 ± 5.5 | |
|  | *(t* = 0.7076, d_f_ = 11, *p* = 0.4939) | | | | |
| Investigation time in the FO1 (2) zone (m) | 15.0 ± 9.9 | | | 16.9 ± 9.7 | |
|  | (*t* = 0.8583, d_f_ = 11, *p* = 0.4091) | | | | |
| Distance travelled in the zone of FO1 (m) | 0.49 ± 0.37 | | 0.61 ± 0.33 | | |
|  | *(t* = 1.287, d_f_ = 11, *p* = 0.2246) | | | | |
| Distance travelled before first investigating in the zone of FO1 (m) | 2.4 ± 2.7 | | 3.1 ± 2.6 | | |
|  | *(t* = 0.00956, d_f_ = 8, *p* = 0.9926) | | | | |
| Latency to first investigation in the zone of FO1 (s) | 80.8 ± 122.2 | | 157.5 ± 165.5 | | |
|  | *(t* = 1.185, d_f_ = 10, *p* = 0.2636) | | | | |
| Average speed while investigating in the zone of FO1 (m/s) | 0.03 ± 0.02 | | 0.04 ±0.01 | | |
|  | *(t* = 0.8174, d_f_ = 10, *p* = 0.4327) | | | | |
| Mobile time spent investigating the zone of FO1 (s) | 15.0 ± 9.9 | | 16.9 ± 9.7 | | |
|  | *(t* = 0.8583, d_f_ = 11, *p* = 0.4091) | | | | |
| Head average distance from the zone of FO1 (m) | 0.4 ± 0.1 | | 0.4 ± 0.2 | | |
|  | *(t* = 1.09, d_f_ = 11, *p* = 0.2989) | | | | |
| Head max distance from the zone of FO1 (m) | 0.8 ± 0.2 | | 0.8 ± 0.1 | | |
|  | W = -34, *p* = 0.2036 | | | | |
| Time spent oriented to the zone of FO1 (s) | 144.9 ± 52.9 | | 217.9 ± 79.1 | | |
|  | *(t* = 2.838, df = 11, *p* = 0.0161*) | | | | |
| NO number investigations | 7.8 ± 7.9 | | 6.7 ± 7.6 | | |
|  | W = -14, *p* = 0.5586 | | | | |
| Investigation time in the NO zone (s) | 12.9 ± 9.9 | | 13.2 ± 18.9 | | |
|  | W = -22, *p* = 0.4238 | | | | |
| Distance travelled in the zone of NO (m) | 0.6 ± 0.5 | | 0.5 ± 0.5 | | |
|  | *(t* = 0.3916, d_f_ = 11, *p* = 0.7028) | | | | |
| Distance travelled before first investigating in the zone of NO (m) | 3.9 ± 4.5 | | 4.2 ± 2.8 | | |
|  | *(t* = 1.506, d_f_ = 7, *p* = 0.1757) | | | | |
| Latency to first investigation in the zone of NO (s) | 168 ± 175 | | 173 ± 186 | | |
|  | *(t* = 0.3316, d_f_ = 9, *p* = 0.7478) | | | | |
| Average speed while investigating in the zone of NO (m/s) | 0.04 ± 0.01 | | 0.05 ± 0.05 | | |
|  | W = -15, *p* = 0.4824 | | | | |
| Mobile time spent investigating the zone of NO (s) | 12.9 ± 9.9 | | 13.2 ± 18.9 | | |
|  | W = -22, *p* = 0.4238 | | | | |
| Head average distance from the zone of NO (m) | 0.6 ± 0.1 | | 0.5 ± 0.2 | | |
|  | *(t* = 0.7682, d_f_ = 11, *p* = 0.4585) | | | | |
| Head max distance from the zone of NO (m) | 0.8 ± 0.1 | | 0.8 ± 0.0 | | |
|  | W = -78, *p* = 0.0005* | | | | |
| Time spent oriented to the zone of NO (s) | 170 ± 60 | | 202 ± 92 | | |
|  | *(t* = 1.594, d_f_ = 10, *p* = 0.142) | | | | |
| Recognition index | 48.1 ± 27.6 | | | | 31.7 ± 20.6 |
|  | *(t* = 2.277, d_f_ = 9, *p* = 0.0488) | | | | |
